# Enclosure and Camouflage Design of a Prototype Remote Monitoring System for the Protection and Conservation of Territories in the Colombian Amazon Rainforest

**DOI:** 10.1002/ece3.73491

**Published:** 2026-04-20

**Authors:** María Barajas, Jorge Torres, Paula Ortiz, Andrés Triana, Margarita Varon

**Affiliations:** ^1^ Department of Electrical and Electronic Engineering Universidad Nacional de Colombia Bogotá Colombia

**Keywords:** 3D‐printing, coatings, Colombian Amazon rainforest, conservation technology, enclosure design, equipment camouflage, housing design, remote monitoring system, setup and mount design, tropical environments, selva amazónica colombiana, recubrimientos, diseño de la carcasa (enclosure design), camuflaje del equipo, sistema de monitoreo remoto, entornos tropicales, tecnología de conservación, diseño de la instalación y del soporte, impresión 3D

## Abstract

Increasing pressure on the Amazon rainforest from illegal mining and deforestation threatens biodiversity. Monitoring such vast and remote territories remains challenging, and the performance of technological monitoring systems largely depends on their ability to withstand harsh environmental conditions and operate unattended for long periods. This article presents the deployment of a Remote Monitoring System (RMS) in the Colombian Amacayacu National Natural Park designed to detect unauthorized entries into the park via waterways. It addresses two critical operational challenges frequently underreported in the literature: equipment protection against environmental conditions and strategies to reduce vandalism or theft. The results demonstrate that appropriate enclosure design, material selection, and camouflage strategies can improve the operational robustness of RMS deployed in humid tropical environments. This experience provides practical design insights and recommendations to support future deployments of long‐term outdoor monitoring technologies.

## Graphical abstract

Enclosure and camouflage design to protect electronic equipment of a Remote Monitoring System used for the conservation of Colombian Amazon Rainforest.

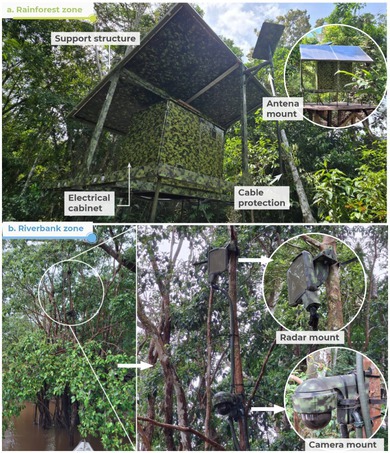

## RESUMEN

La creciente presión sobre la selva amazónica, provocada por la minería ilegal y la deforestación, amenaza la biodiversidad. La vigilancia de territorios tan vastos y remotos sigue siendo un desafío, y el rendimiento de los sistemas tecnológicos de vigilancia depende en gran medida de su capacidad para soportar condiciones ambientales adversas y funcionar sin supervisión durante largos períodos. Este artículo presenta la implementación de un sistema de vigilancia remota (RMS) en el Parque Nacional Natural Amacayacu de Colombia, diseñado para detectar entradas no autorizadas al parque a través de vías fluviales. Aborda dos desafíos operativos críticos que con frecuencia no se mencionan en la literatura: la protección del equipo contra las condiciones ambientales y las estrategias para reducir el vandalismo o el robo. Los resultados demuestran que un diseño adecuado de la carcasa (enclosure design), la selección de materiales y las estrategias de camuflaje pueden mejorar la robustez operativa de los RMS desplegados en entornos tropicales húmedos. Esta experiencia proporciona conocimientos prácticos de diseño y recomendaciones para respaldar futuros despliegues de tecnologías de monitoreo al aire libre a largo plazo.

## 1 Introduction

The Colombian Amazon rainforest is recognized as one of the most biodiverse ecosystems on the planet. However, the region is facing increasing threats, including illegal mining and deforestation (Amazon Conservation Team 2025; Organización de las Naciones Unidas para la Educación, Ciencia y la Cultura (UNESCO) 2025). Monitoring such vast and remote territories to detect these activities remains one of the greatest challenges for conservation authorities.

The park rangers of Colombia's National Natural Parks (CNNP) monitor threats in these areas, using different strategies, such as river patrols, camera traps, overflights, and satellite monitoring (Ministerio de Medio Ambiente y Desarrollo Sostenible, Parques Nacionales Naturales de Colombia, and República de Colombia 2021). Modern monitoring techniques utilizing Geographic Information Systems (GIS) and sensing have proven to be robust tools for monitoring environmental and territorial dynamics, as demonstrated by studies on long‐term forest changes (Valjarević et al. 2018), as the detection of harvested forest areas in Italy (Borrelli et al. 2014), and structural alterations in river basins (Valjarević 2024). Nevertheless, these approaches rely mainly on data processed over extended timeframes, preventing the generation of real‐time alerts required by the authorities to enact timely mitigation strategies during critical events. This has led to the development of new alternatives such as Remote Monitoring Systems (RMS). RMS are designed to collect and transmit information, such as photographs, audio, or video automatically in remote regions (Zwerts et al. 2021). These systems have been implemented in various locations around the world (Torres Cepeda et al. 2025) and in the Amazon rainforest (HUAWEI 2023; Rainforest Connection 2023; Rodrigues et al. 2025). Nevertheless, their long‐term performance in the field, outdoors and unattended, poses significant challenges: first to withstand environmental conditions with an enclosure design, referring to the physical pieces of hardware necessary to install and protect the equipment; and second to mitigate the risk of vandalism or theft.

Equipment failure or loss interrupts monitoring programs, causing data gaps and generating high replacement and logistical costs (Meek et al. 2019). Although conservation practitioners adopt mitigation strategies to deal with insects, moisture, thieves, and humidity among others, their solutions aren't documented in detail, making replication difficult (Glover‐Kapfer et al. 2019). Specifically, for tropical rainforests, this issue is rarely addressed in the literature, such as Lascaro (1970), that describes equipment protection and design techniques based on his experience in Southeast Asia, and Miranda et al. (2000), who focus on corrosion protection for radar towers used in the Amazon Surveillance System (SIVMA). However, these cases lack sufficient detail regarding the practical application of their recommendations and do not consider camouflage strategies.

Therefore, this paper presents the design, implementation, and installation of an RMS adapted to the Colombian Amazon rainforest ecosystem conditions. The proposed system employs a hybrid architecture, integrating satellite communications with mission‐oriented sensors. Within this framework, this article focuses particularly on the design of the mounting and housing components, including the selection of materials, coatings, and colors intended to slow down equipment deterioration and to reduce its visibility through a camouflage strategy. At the time of writing this article, the installed prototype had been in operation for eight months in a test area within the Amacayacu National Natural Park (ANNP). While the system described here was developed for a specific deployment in the Colombian Amazon, several of the design considerations derived from this experience represent practical principles that may be helpful for the development of monitoring systems in other remote conservation settings. The main insights from this experience are presented here to serve as a guide for the deployment of monitoring stations in tropical rainforests. This study focuses primarily on the operational deployment of the system; the evaluation of its long‐term conservation effectiveness, such as its influence on illegal activity detection or deterrence, is beyond the scope of this article.

## 2 Methods

**FIGURE 1:**
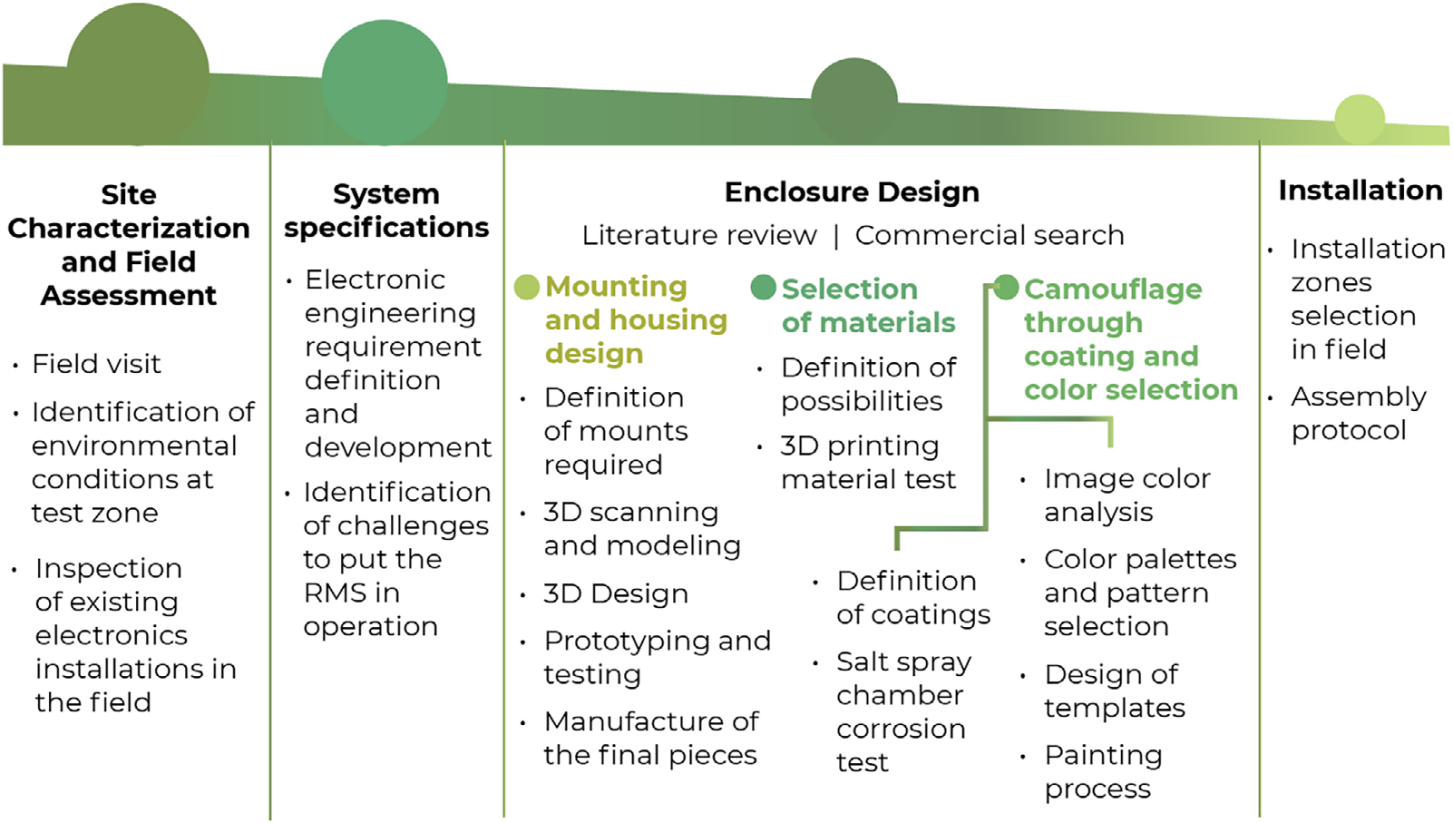
Stages in the development process of the remote monitoring system (RMS) prototype. The diagram illustrates the sequential workflow followed for the design and implementation of the RMS, including site characterization and field assessment, definition of system specifications, enclosure design, and final installation. The highlighted stages correspond to the three main challenges addressed in this study: Mounting and housing design, material selection, and camouflage through coatings and color definition.

This methodology encompassed the process for designing, adapting, and installing the RMS (Figure 1). This article focuses on the three key challenges identified during the research: mounting and housing design, selection of materials, and camouflage through coating and color selection. The technical and electronic design is beyond the scope of this publication. Although the methodological process was developed for a specific ecological and logistical context, the design workflow presented here may provide a transferable framework for developing technological monitoring systems in other remote or environmentally challenging conservation areas.

### 2.1 Site Characterization and Field Assessment

The development of the RMS began with an exploratory visit to the ANNP (3°50′‐3°02′ S, 69°54′‐70°20′ W), a park in southern Colombia chosen as a pilot area due to its representative warm tropical rainforest and seasonal floodplain forest ecosystems. The park experiences an average annual rainfall of 2836 mm, temperatures ranging from 26°C to 32°C, and a relative humidity of 90%. The level of the Amazon River varies from 81 m in winter to 55 m in summer (Parques Nacionales Naturales de Colombia 2025).

**FIGURE 2:**
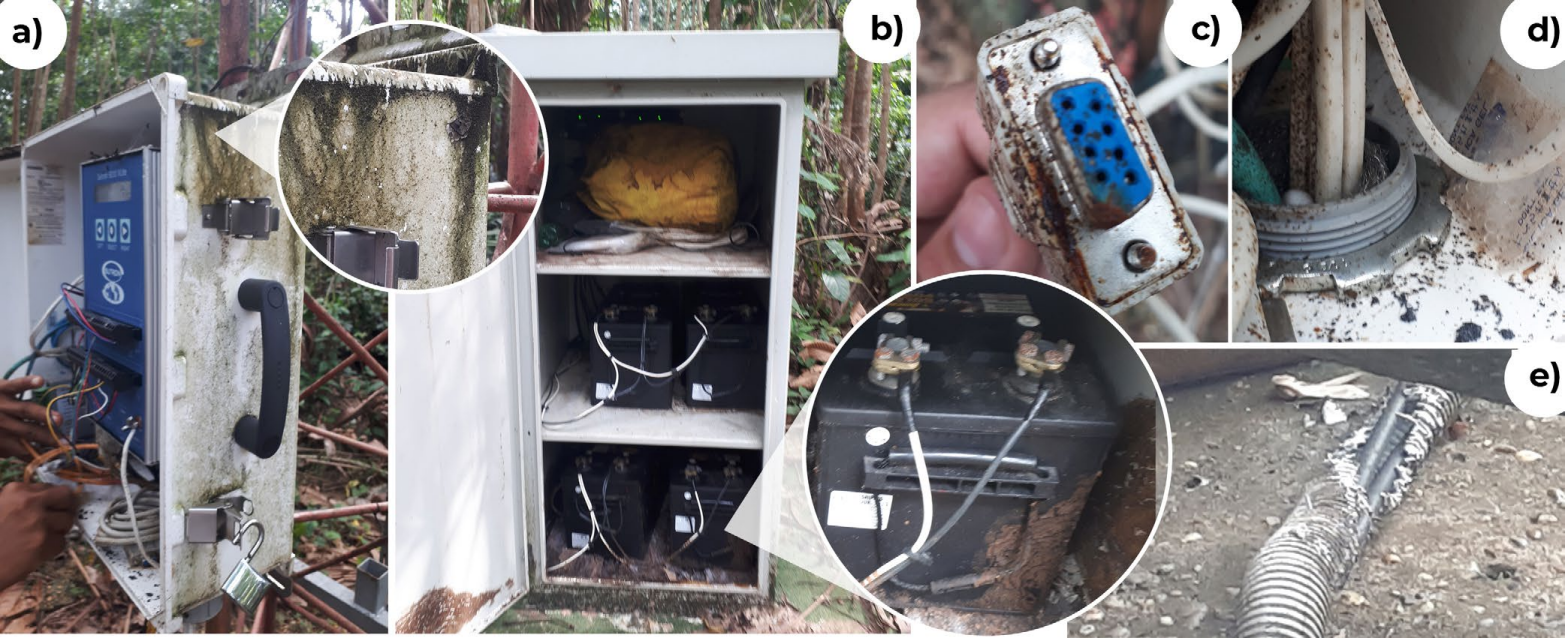
Design failures identified during the field visit to a climatological monitoring tower in Amacayacu National Natural Park (ANNP). Photographs documenting deterioration mechanisms observed in existing outdoor infrastructure: Corrosion of metallic components (a–c), sealing failures and insect colonization (d), and cable damage likely caused by fauna (e). These field observations informed the enclosure and protection criteria adopted for the Remote Monitoring System (RMS).

During the visit, interviews with local inhabitants and park rangers explored their expectations for the RMS and their past experiences with electronic equipment. Fieldwork also included inspecting existing installations: off‐grid photovoltaic systems, satellite dishes, Ultra High Frequency (UHF) radios, and a climatological monitoring tower. The latter was found out of service due to component deterioration from the rainforest's extreme conditions (Figure 2).

After the visit, the following considerations for the design and installation of equipment were identified:

- Metal components were found to be susceptible to corrosion (Figure 2a–c).
- High temperature generated by equipment attracted insect colonies (Figure 2d).
- Sealing failures caused water condensation and insect entry (Figure 2d).
- Cables were at risk of rodent‐inflicted damage (Figure 2e).
- Photovoltaic modules developed fungi when water was stagnant.
- Wildlife, including monkeys, birds, and insects, represented another risk to the equipment.
- High rainfall necessitated the use of suspended or elevated structures.
- Electrical and telecommunications networks were absent.

### 2.2 System Specifications

The minimum requirements for implementing an RMS in the Colombian Amazon rainforest were defined as: energy autonomy and internet connectivity, the prototype had to incorporate an autonomous energy supply system, and network access needed to be ensured to send the collected information; likewise, ease of handling was required during installation, operation, and maintenance. Regarding durability, robust and resistant materials were needed to ensure reliable long‐term performance; finally, camouflage requirements needed to be implemented to minimize visual impact on the ecosystem and avoid detection by potential human intruders.

In the Colombian Amazon, illegal dredging activities access protected forest areas primarily through river corridors. Therefore, monitoring the river is not an independent objective, but a strategic approach to controlling access routes to natural parks and adjacent forest ecosystems. By detecting and tracking vessel movements along the river, the system functions as an early warning mechanism for potential illegal extraction activities that may impact surrounding forest environments. Based on the defined requirements, the RMS was designed to autonomously detect boat entries in remote areas of the Colombian Amazon, monitoring river headwaters, identifying vessels, and sending real‐time alerts to park rangers via e‐mail with photos. It consisted of three integrated modules:

The detection module incorporated a SpotterRF CK5‐CE compact radar operating at 24 Gigahertz (GHz), with a spatial resolution of 1 m and a detection range of up to 175 m for river vessels. The radar is capable of tracking multiple moving targets simultaneously and provides positional and velocity information. The system was integrated with a Pan–Tilt–Zoom (PTZ) network camera with 360° horizontal rotation, 180° vertical rotation, and up to 10 × optical zoom, enabling automated visual tracking of detected targets. A Networked Input/Output (NIO) device coordinated the interaction between radar and camera, enabling event‐triggered image capture and automated alert generation (Cepeda et al. 2026; Torres Cepeda 2023).

The telecommunications module was based on a commercial satellite communication system, Starlink, operating in the Ku band with typical link speeds between 40 and 220 Megabits per second (Mbps) and latency below 99 milliseconds (ms). The system included a satellite antenna and a router, enabling internet connectivity in the absence of terrestrial communication infrastructure (Cepeda et al. 2026; Torres Cepeda 2023).

The autonomous power supply module consisted of a 48 Voltage Direct Current (VDC) off‐grid photovoltaic system designed to supply an estimated average load of approximately 120 W (W). The system incorporated two 580 W monocrystalline photovoltaic panels connected in series, a Victron Energy SmartSolar MPPT 150/45 charge controller, and a Victron Energy Phoenix 48/375 VE. Direct inverter (120 VAC output), two Pylontech US5000 lithium‐ion batteries (48 VDC, 4800 Wh nominal capacity each), and a Victron Energy Cerbo GX energy management system for remote monitoring of power generation, storage, and consumption parameters (Cepeda et al. 2026; Torres Cepeda 2023).

### 2.3 Enclosure Design

To put the RMS into operation in the field, three design challenges were identified: the mounting and housing design, which required each electronic component to be properly positioned and protected from environmental conditions; the selection of durable, weather‐resistant materials to protect against physical deterioration of the mounting and housing elements; and camouflage through coating and color selection, carefully chosen to minimize visual detection and the risk of vandalism.

#### 2.3.1 Mounting and Housing Design

Based on the site's environmental characteristics of the ANNP, the setup was distributed across two different zones: the detection module was installed near the riverbank, while the telecommunications and photovoltaic modules were placed 70 m inland from the riverbank, hidden among the vegetation. Cables interconnecting both zones demanded robust protective conduits (cable protection) to prevent damage from moisture, wildlife, and mechanical stress. Each zone required a customized outdoor setup explained below.

Riverbank zone: where the camera and radar were attached to a tree trunk at a height of eight meters to improve the line‐of‐sight and reduce the risk of flooding. This zone included a camera mount and a radar mount to hold the camera and the radar, and to host its cables, its cable joints, and connectors for cable protection. Although many commercial mounting options existed, they were discarded due to the significant modifications required to fit the cable protection and to host the cable joints needed for installation. As a result, custom mounts were designed and manufactured using 3D printing (discussed in detail later).

Rainforest zone: where the solar panels, the antenna, and other electronic components remained slightly elevated from the ground, requiring minimal obstructions to the sky to achieve direct exposure to sunlight and satellite signals. This zone included an electrical cabinet to host and protect electronic equipment for control, distribution, and regulation of the three modules. The cabinet was required to comply with the IP66 rating (Ingress Protection Rating), according to the IEC (International Electrotechnical Commission) standard 60,529, ensuring hermetism against dust and high‐pressure water jets (International Electrotechnical Commission 2025). The zone also incorporated a support structure to maintain the electrical cabinet elevated above the ground and to hold the solar panels at a fixed 15° angle to optimize energy capture and facilitate self‐cleaning. In addition, an antenna mount was necessary to ensure adequate stability and height of the antenna.

To design the setup parts, 3D models were developed based on accurate dimensional measurements. Design proposals were prototyped to validate their fit, functionality, and assembly procedures.

The design of all setup elements followed these key criteria:

- Water drainage: Surfaces were shaped to allow rainwater to flow off rather than accumulate or enter. In addition, mechanical unions were protected to avoid contact with water.
- Insect resistance: All enclosures were designed to prevent the ingress of biological agents such as insects or moisture.
- Visual integration: Discreet contours and finishes were chosen to minimize visual impact and avoid drawing attention.
- Lightweight yet durable: Materials were selected for strength and low weight to facilitate transport and structural integrity.
- Modularity and reparability: Components were designed using several pieces to enable partial replacements in case of damage.
- Redundant mounting systems: Mounts feature three attachment mechanisms to ensure secure installation on tree trunks.
- Adaptability to various tree diameters: Flexible mounting configurations were implemented to accommodate different trunk sizes.
- Ease of transport and installation: Parts were modular and compact to simplify logistics.

#### 2.3.2 Material Selection

To manufacture the mounts and housing elements, several methods and materials were considered. Given the low‐volume production required, 3D‐printing was selected for the camera and radar mounts, as a fast and cheap manufacturing method that builds realistic and accurate pieces without molds. To select the 3D‐printing material, two experimental tests were conducted using PLA (Poly Lactic Acid), PP (Polypropylene), ASA (Acrylonitrile Styrene Acrylate), resin (Hydrophilic group‐modified light‐curing), and PETG (Polyethylene Terephthalate Glycol‐Modified). These materials were selected due to their degradability, mechanical compression, and tensile properties (Ramírez‐Revilla et al. 2023) following the standards D695‐02a (American Society for Testing and Materials (ASTM) 2026a) and D638‐14 (American Society for Testing and Materials (ASTM) 2026b).

In the first test, one sample of each 3D‐printing material measuring 100 × 150 × 2 mm was exposed outdoors for nine months to evaluate its resistance to sunlight and water. The samples exposed were photographed before and after, measured, and compared with a non‐exposed sample. Deformation, buckling, fungal growth, and decomposition were documented; according to the results, low‐performance materials were discarded. In the second test, to evaluate dimensional stability and printability, a camera mount piece was 3D‐printed with the remaining materials to assess their distortion compared to an ideal 3D model of the same piece. The finish quality, the time consumed, and the ease of printing were documented.

Cold‐rolled metal sheet was used for the pieces supporting the structure, the electrical cabinet, and the antenna mount, to meet the requirements of standards for electrical cabinets (Ministerio de Minas y Energía and República de Colombia 2025) and photovoltaic structures (International Electrotechnical Commission (IEC) 2023). As a corrosion inhibitor, standards suggested galvanized coatings. However, a study evaluating corrosion resistance of galvanized sheets in tropical environments (Xenia Isbel and René Valentino 2014), warned that in highly corrosive environments galvanized coatings could be insufficient. Therefore, two corrosion inhibitors were tested in a salt spray chamber (discussed in detail in the next sections).

#### 2.3.3 Camouflage Through Coatings and Color Selection

##### 2.3.3.1 Coatings

As a camouflage strategy, coatings were selected to create a multicolor mottled pattern applied to the mounting and housing elements. Two alternatives were considered and tested: painting or applying a printed film.

Regarding metal pieces, they had to be coated with electrostatic paint, which should be applied uniformly and required a curing process (Schrader and Elshennawy 2000) inhibiting the multicolored pattern. Therefore, the camouflage pattern had to be created using an additional layer applied over this base coat.

In the absence of specific evidence on the behavior of the paints and coatings under the conditions of the Amazon rainforest, an experimental test was conducted. High humidity was identified as a major concern, as it could cause coating detachment and accelerated degradation. Therefore, a salt spray chamber test was conducted on samples covered with different coatings selected according to literature sources, expert advice, and field observations. The test pursued four main objectives:

- To visualize potential material damage without corrosion protection: using a cold‐rolled sheet with no coating.
- To determine the most effective corrosion inhibitor: using one cold‐rolled sheet with a galvanized coating and another with anti‐corrosive paint.
- To identify the best coating for the camouflage layer: using cold‐rolled galvanized sheets coated with electrostatic paint and various types of paints: epoxy, lacquer, polyester, polyurethane, enamel, and graffiti‐type paint for option one; and camouflaged car‐wrapping vinyl and camouflaged water‐transfer printing film for option two.
- To determine the best alternative for plastic components: using sample sheets coated with graffiti‐type paint and water‐transfer printing, the latter was also tested on cable protection.

The experiment was conducted using the Atlas SF Corrosion Exposure System in accordance with ASTM B117‐25 (American Society for Testing and Materials (ASTM) 2025). The samples were exposed to a salt spray of 5% NaCl at 35°C and 90% relative humidity for 100 h. The samples were photographed before and after exposure to detect rust, detachment, peeling, wrinkles, cracking, and loss of gloss, and finally, to find the sample with fewer alterations after the experiment. Other aspects such as ease of application, color availability, technology required, water consumption, and cost were also considered.

##### 2.3.3.2 Color Selection

To select the colors, reference photographs taken at ANNP were analyzed to generate eight color palettes, each containing five colors that visually blended with the surrounding environment. The photos were intentionally pixelated using software to reduce the color options and to identify redundancies. The sky color and other non‐representative colors were discarded. The final selection aimed to find in each analyzed photo the darkest color, the most vivid color, and three other different colors frequently found in the most relevant elements of the environment, in this case, shades of green and brown from trees, mud, leaves, and the ground. The color palette options were superimposed over the original photos to test coherence; then the palettes were adjusted based on the commercial availability of the colors and evaluated by the park rangers.

For the painting process, a pixelated mottled pattern was adapted into four laser‐cut stencil templates from polypropylene sheets, excluding the background color; each stencil fit one color. To ensure accurate scaling, the pixelated mottled pattern in different sizes was printed on paper and observed at different distances (5, 10, 50, and 100 m).

After the experiments, the mounting and housing elements were coated in a controlled urban environment. To reduce the visibility of the photovoltaic panels from a distance, their white undersides were covered with plastic sheets that were also coated. As a final coating layer, an automotive matte varnish was applied, as it provided strong resistance against UV rays, humidity, and rainfall.

### 2.4 Installation

Once the design stage was finished, the RMS was ready to be installed at ANNP. The assembly protocol consisted of transporting the RMS setup to the ANNP headwater, selecting and clearing the two required zones to ensure the correct equipment operation, and assembling, securing, powering, and testing the system.

## 3 Results

### 3.1 Mounting and Housing Design Results

**FIGURE 3:**
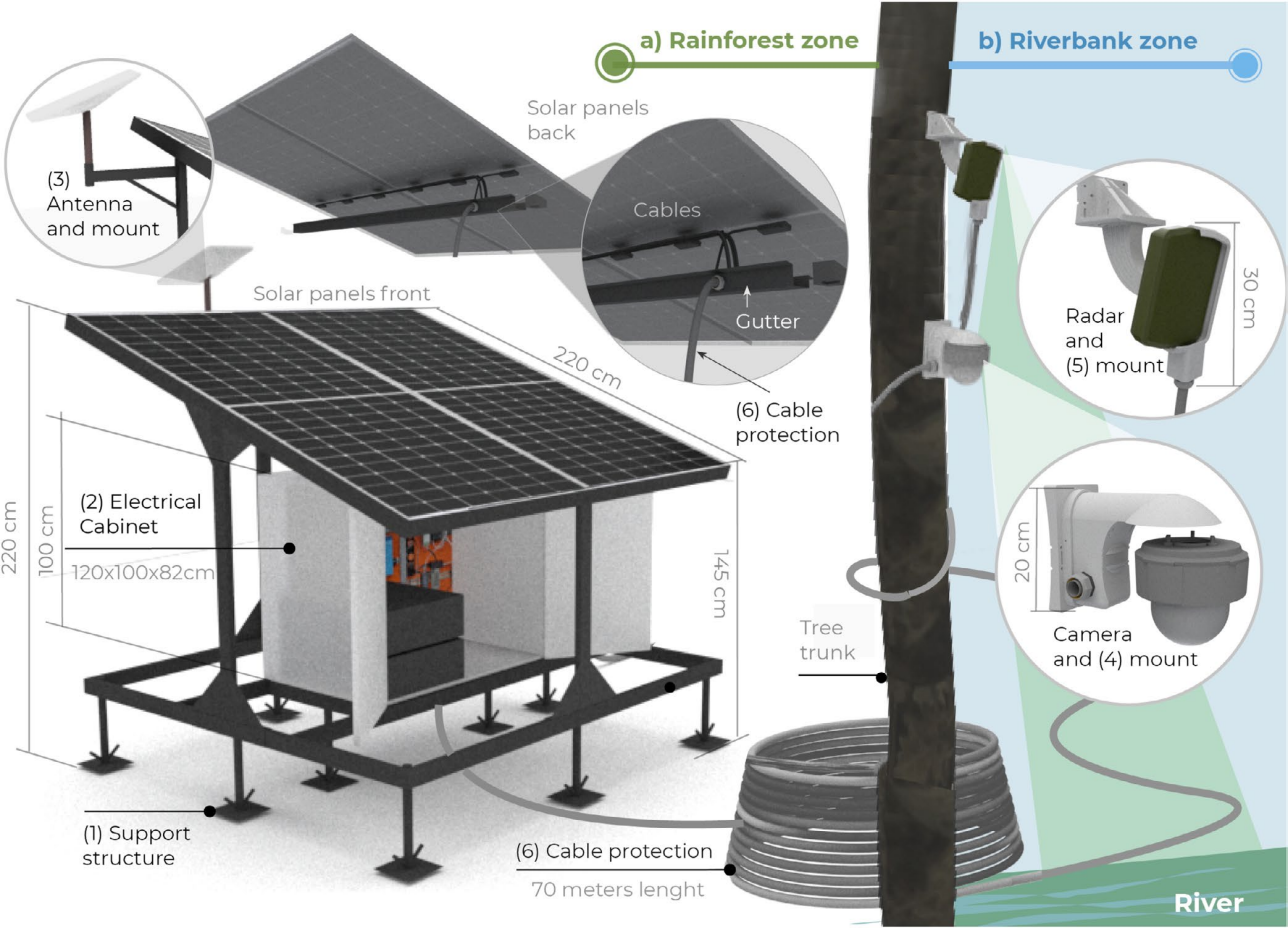
Mounting and housing configuration of the Remote Monitoring System (RMS) prototype. (a) Rainforest zone, showing the modular support structure (1), elevated electrical cabinet (2), antenna mount (3), and integrated cable protection system (6). (b) Riverbank zone, showing the camera mount (4) and radar mount (5) manufactured using 3D‐printing, fixed to the tree trunk. The layout illustrates the two‐zone installation strategy adopted.

At the rainforest zone (Figure 3a), the support structure (1) designed as a modular system, consisted of two horizontal frames: the upper one for mounting the solar panels and the lower one for keeping elevated off the ground the custom‐made electrical cabinet (2) through 10 legs. The frames were spaced apart by four vertical columns, with the front and back differing in height to create the 15° inclination necessary for the solar panel's operation. At the highest column, the antenna mount (3) was attached at the maximum height. A custom gutter beneath the solar panels was implemented to house their cables, protecting them from insects and rodents. The gutter, the antenna mount, and the electrical cabinet were adapted to integrate the cable protection system (6).

A Liquid‐tight Flexible Metal Conduit (LFMC) (6) was used to shield the cables connecting components inside the electrical cabinet to those located outside: the gutter and the mounts of the antenna, the camera, and the radar. Some conduits reached lengths up to 70 m.

**FIGURE 4:**
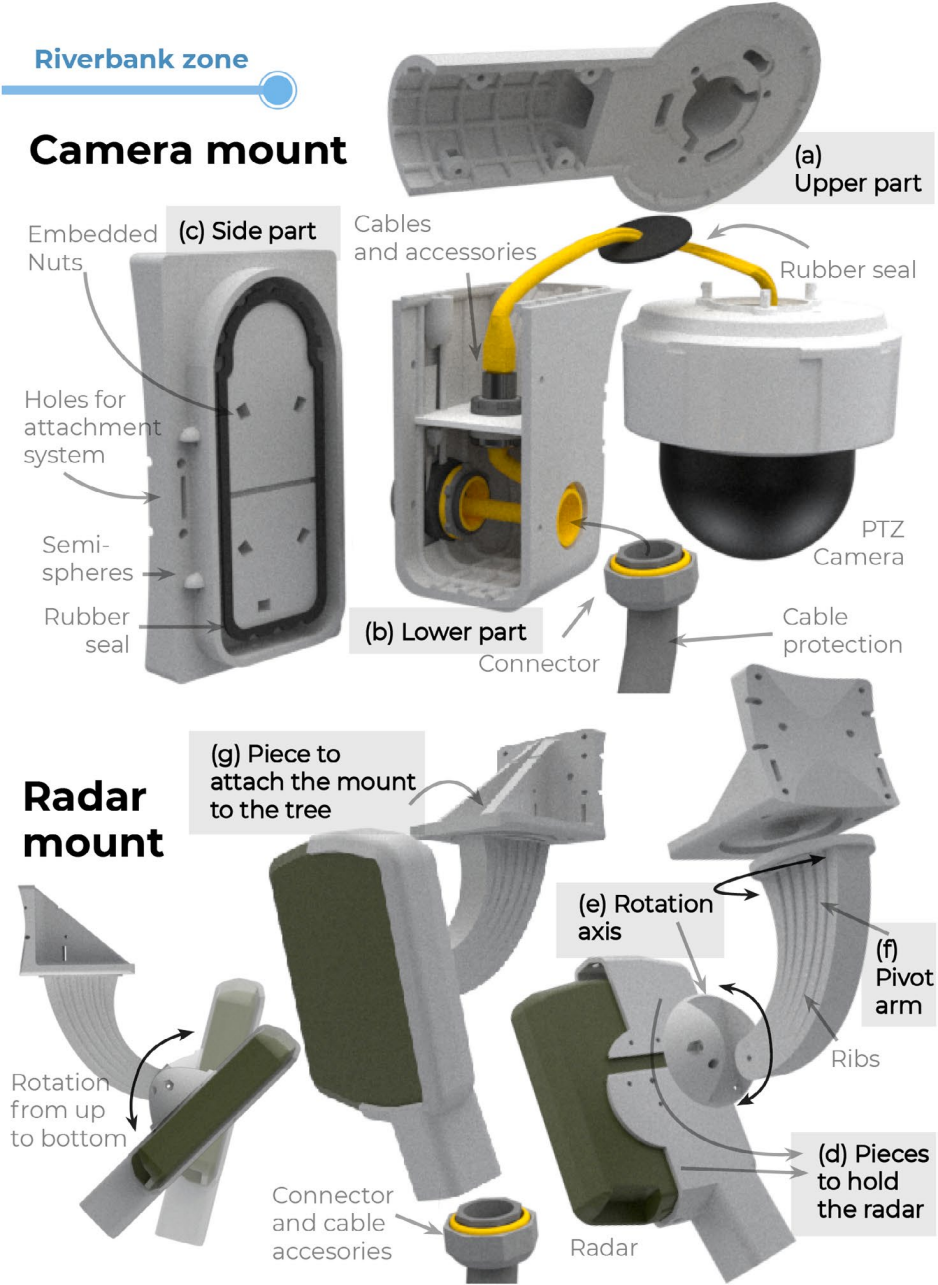
Detailed design of the 3D‐printed camera and radar mounts. Camera mount components include upper (a), lower (b), and lateral (c) pieces forming a hermetic enclosure. Radar mount components include radar holder (d), vertical rotation axis housing (e), pivoted arm (f), and trunk attachment base (g). Internal ribs, embedded nuts, sealing elements, and modular assembly features are shown to illustrate structural reinforcement and water ingress prevention.

To support the elements in the riverbank zone (Figure 3b), the camera (4) and radar (5) mounts were manufactured using 3D‐printing. These mounts had an internal structure of vertical and horizontal ribs to create strong yet lightweight components. Each mount was designed as a set of several custom‐designed parts (Figure 4):

The camera mount enabled the camera to have a wide line of sight while maintaining internal hermetic sealing through a half‐wood joint on its three pieces (a,b,c). The upper part (a) held the camera from above and included rubber seals at the cable outlets to prevent camera fogging; the lower part (b) housed the cable accessories; and the side part (c) closed the mount and attached it to the tree trunk. To assemble the pieces, nuts were embedded inside the upper (a) and side parts (c), and screws crossed the walls to reach them. The head screws were protected from water by semi‐spherical covers that functioned as umbrellas.

Regarding the radar mount, it was designed to facilitate the radar pointing with a pivoted arm (f) that rotated 180° from left to right and from top to bottom. Screws and embedded nuts locked the radar in the desired position. Five pieces constituted the mount: two to hold the radar (d) and its cable accessories, with rubber seals to prevent water ingress; one to house the metallic vertical rotation axis (e); one pivoted arm (f); and one base to house the horizontal rotation axis (f) and to attach the entire mount to the tree trunk (g).

The parts in direct contact with the tree trunk (c and g) for both mounts were designed with a semi‐curved surface to accommodate different trunk diameters. To attach the mounts to the trunk, three different attachment methods could be used: a metallic trail lock, PVC‐covered wire rope, and zip ties.

### 3.2 Test Results to Select Materials, Camouflage Coatings, and Colors

Table 1 presents the results of the 3D printing material samples under the outdoor exposure test with photos of the initial condition and the final condition after nine months.

**TABLE 1:** Results of 3D printing material samples under outdoor exposure test. Comparison of PLA (Poly Lactic Acid), PP (Polypropylene), ASA (Acrylonitrile Styrene Acrylate), resin (hydrophilic group‐modified light‐curing), and PETG (Polyethylene Terephthalate Glycol‐Modified) based on nine‐month outdoor exposure evaluating deformation, discoloration, fungal growth, and structural stability.

	PLA (Poly Lactic Acid)	PP (Polypropylene)	ASA (Acrylonitrile Styrene Acrylate)	Resin (Hydrophilic group‐modified light‐curing)	PETG (Polyethylene Terephthalate Glycol‐Modified)
Initial condition	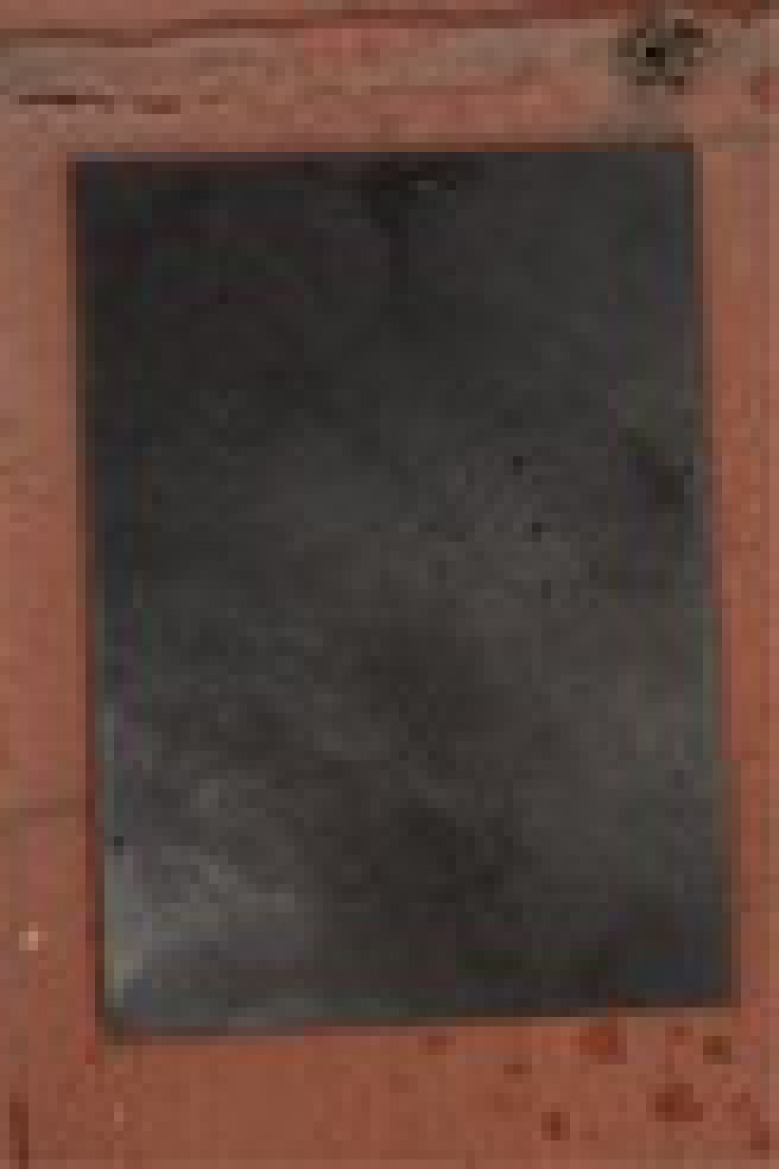	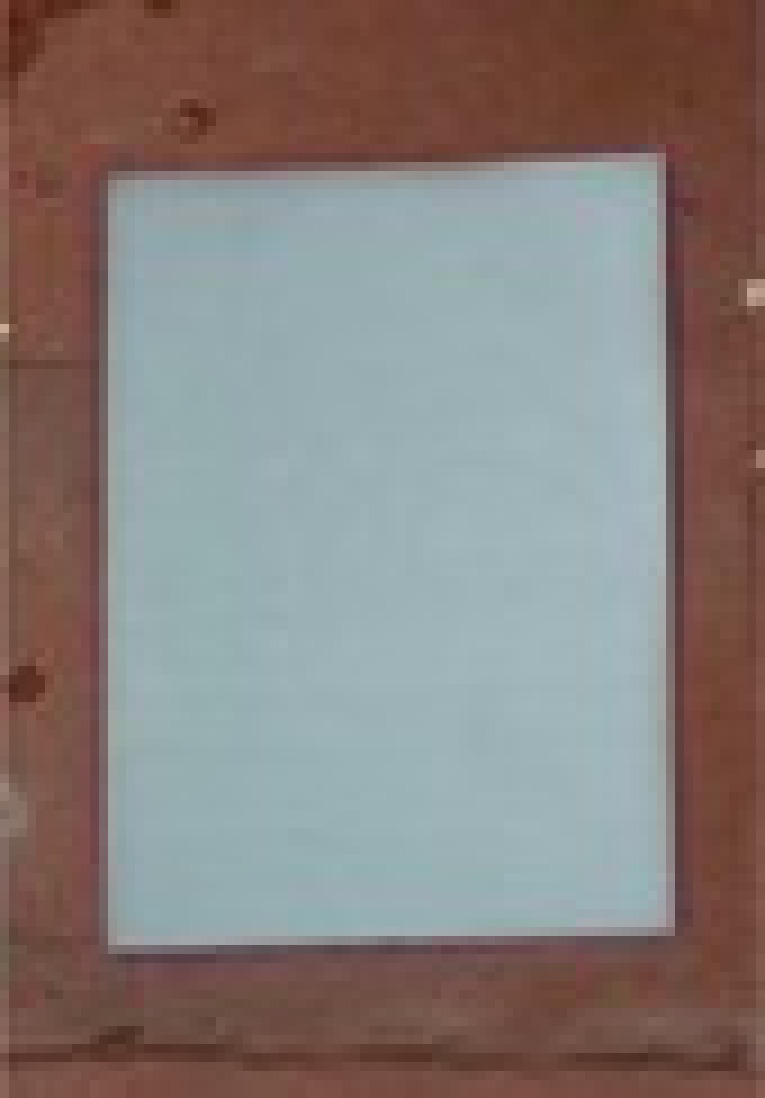	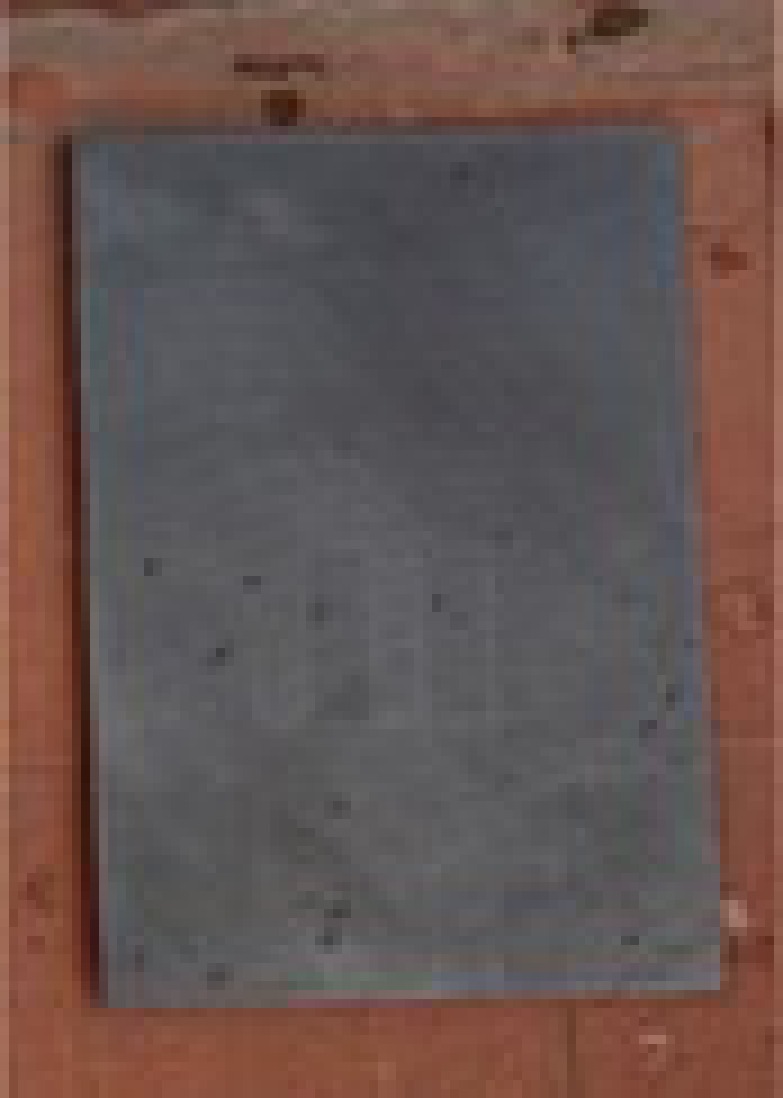	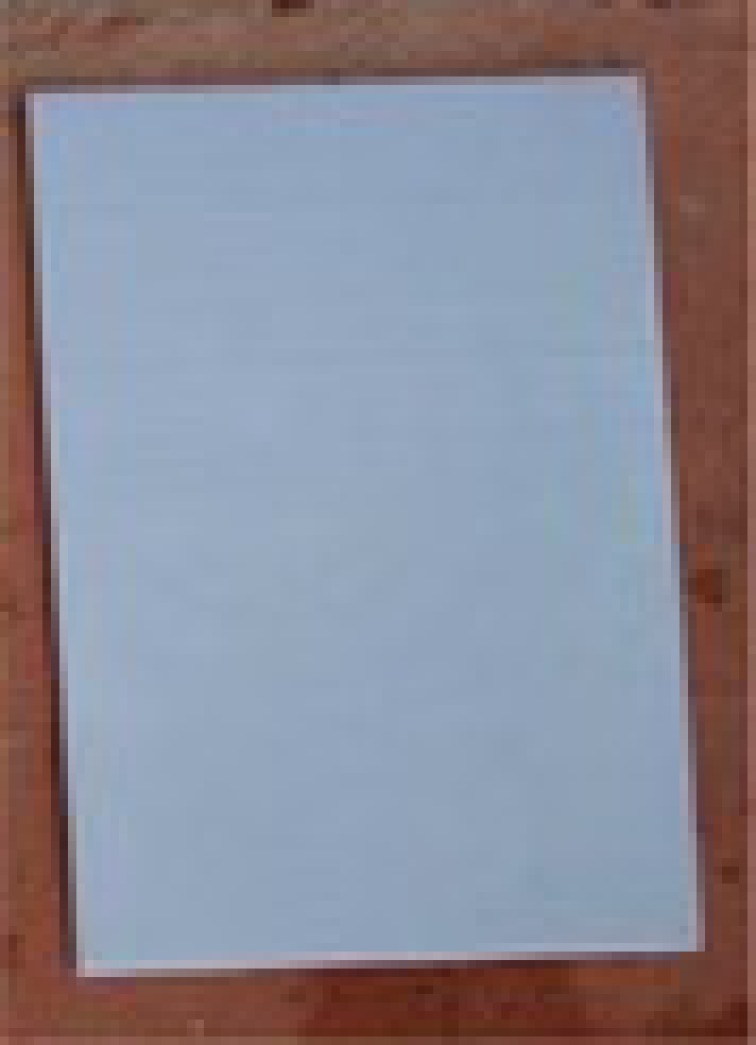	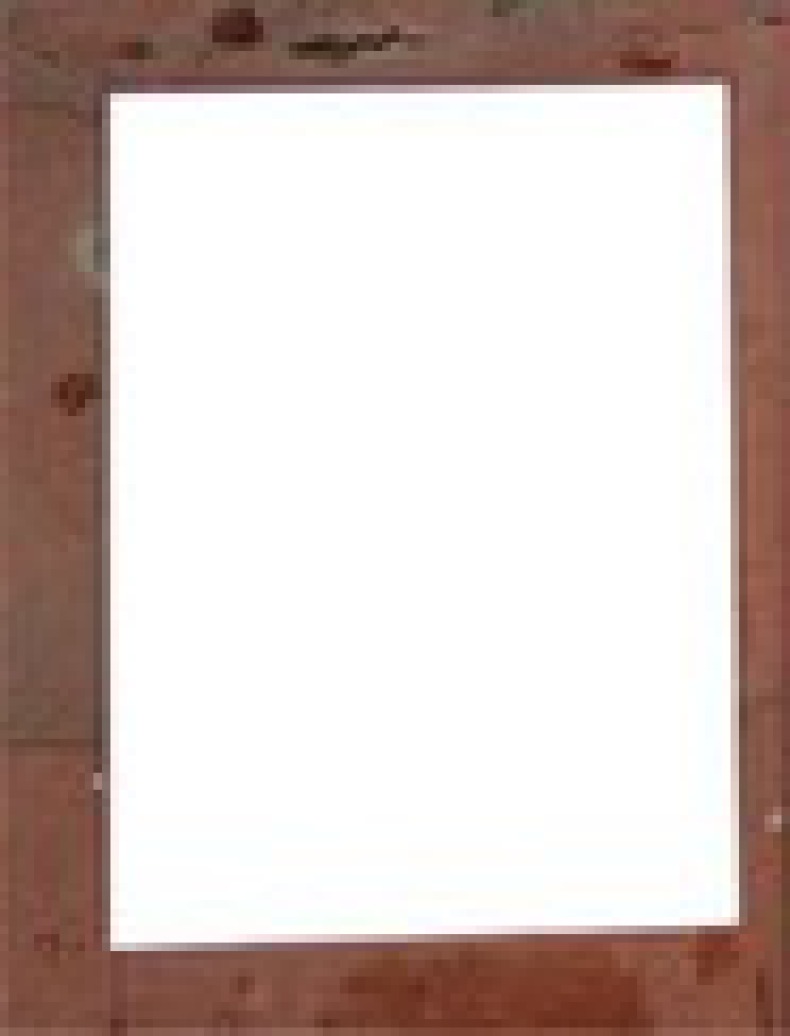
Final condition	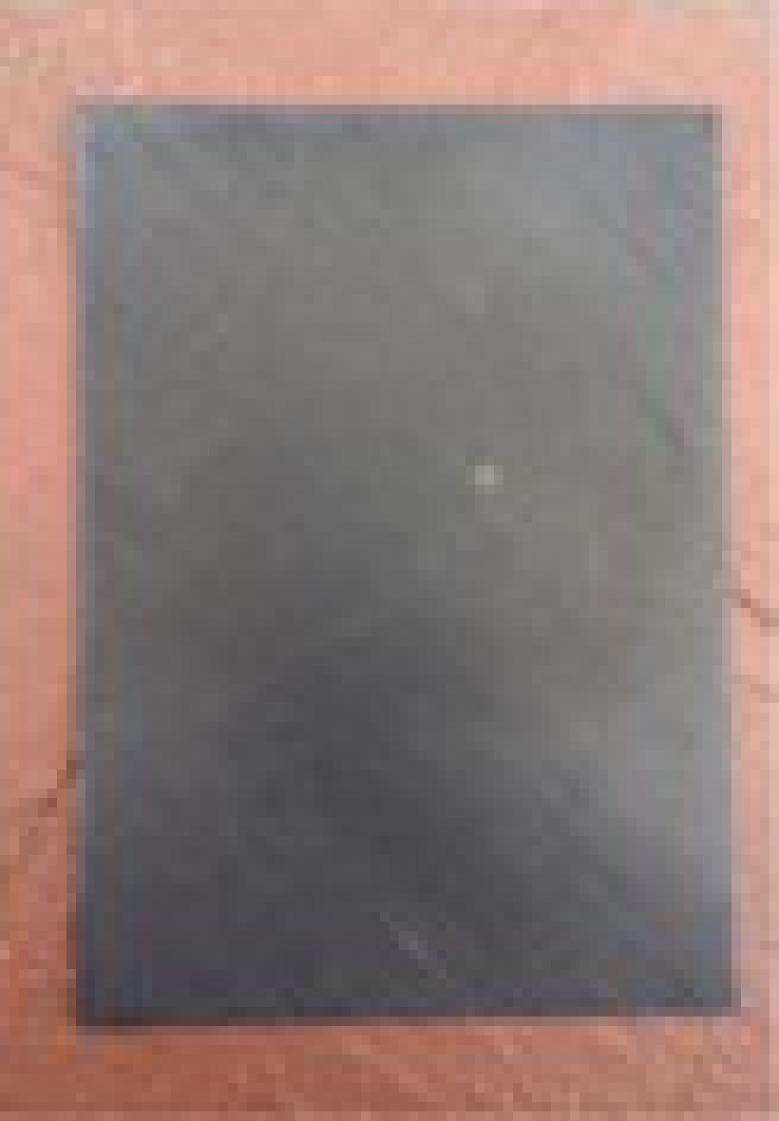	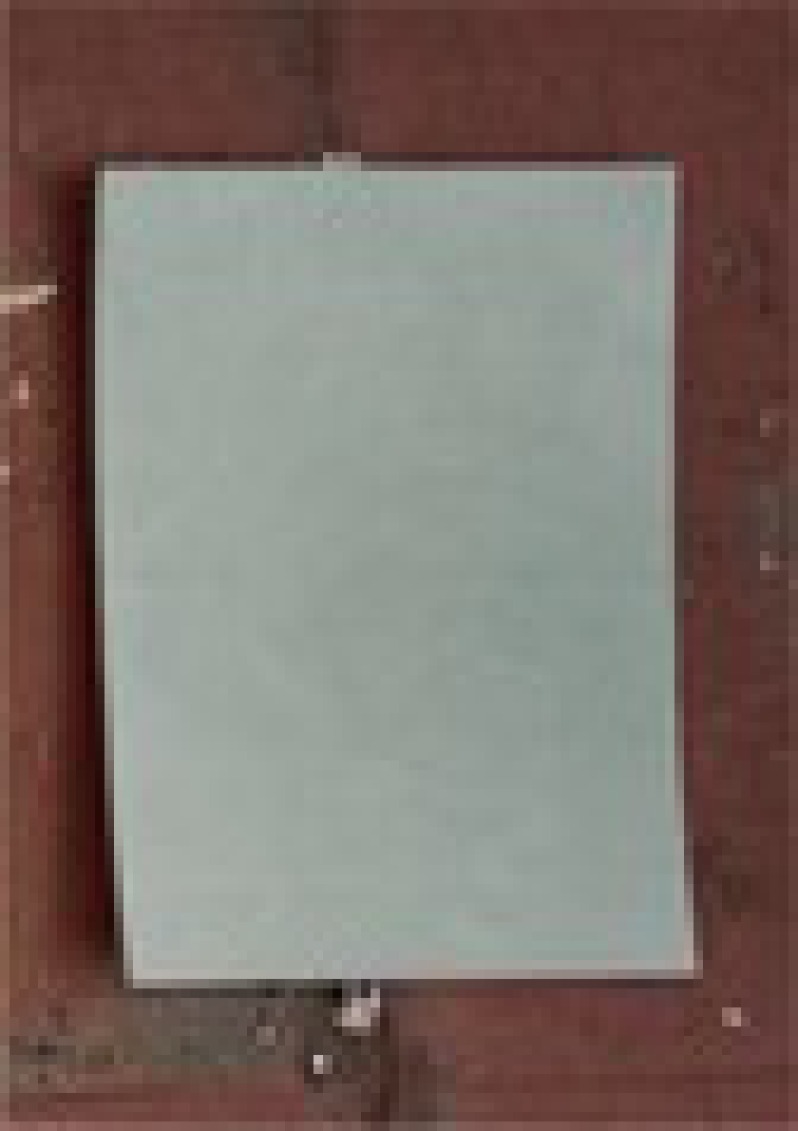	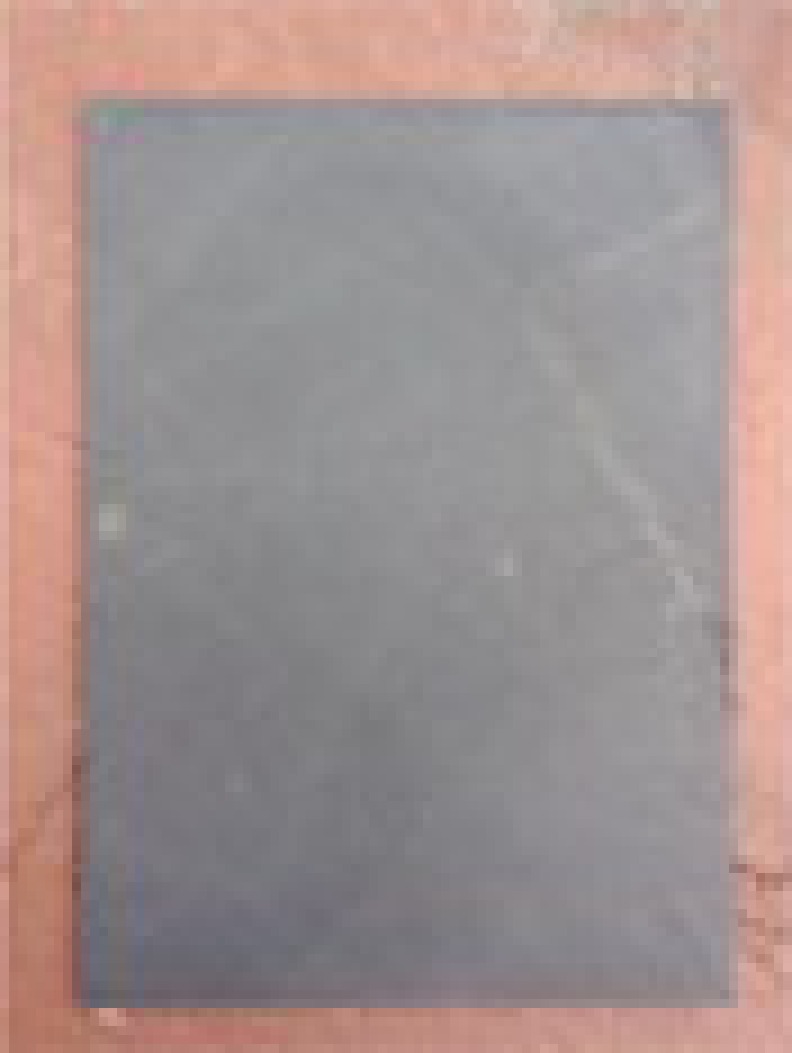	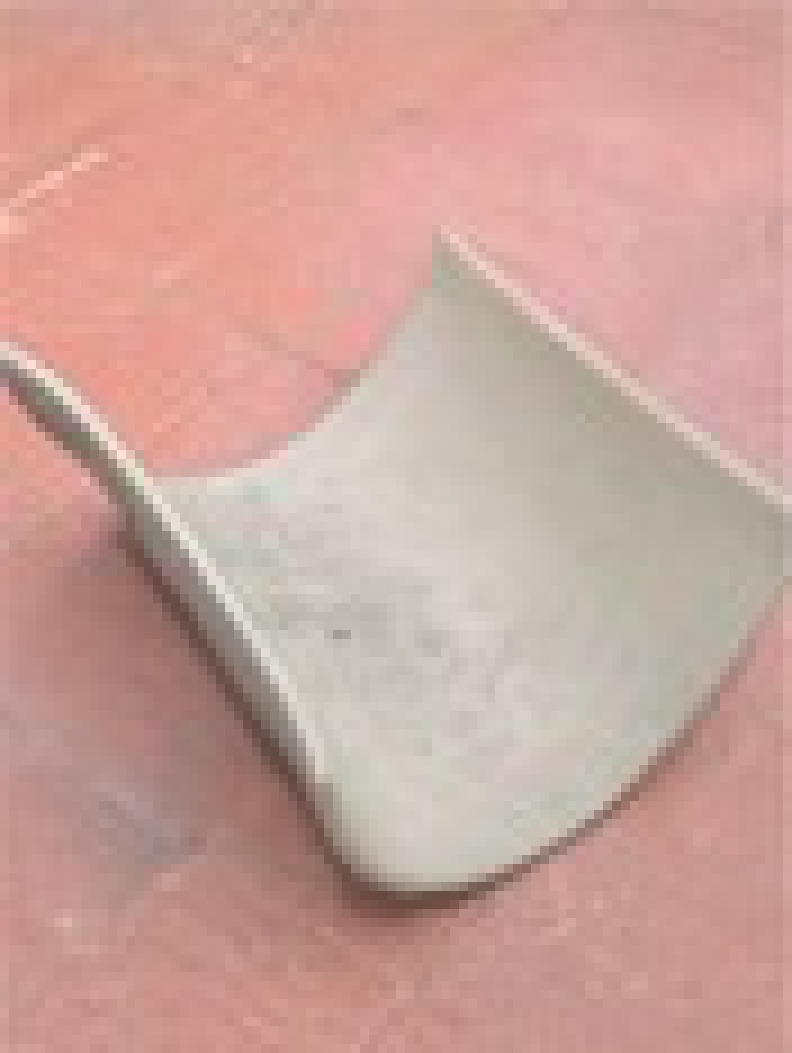	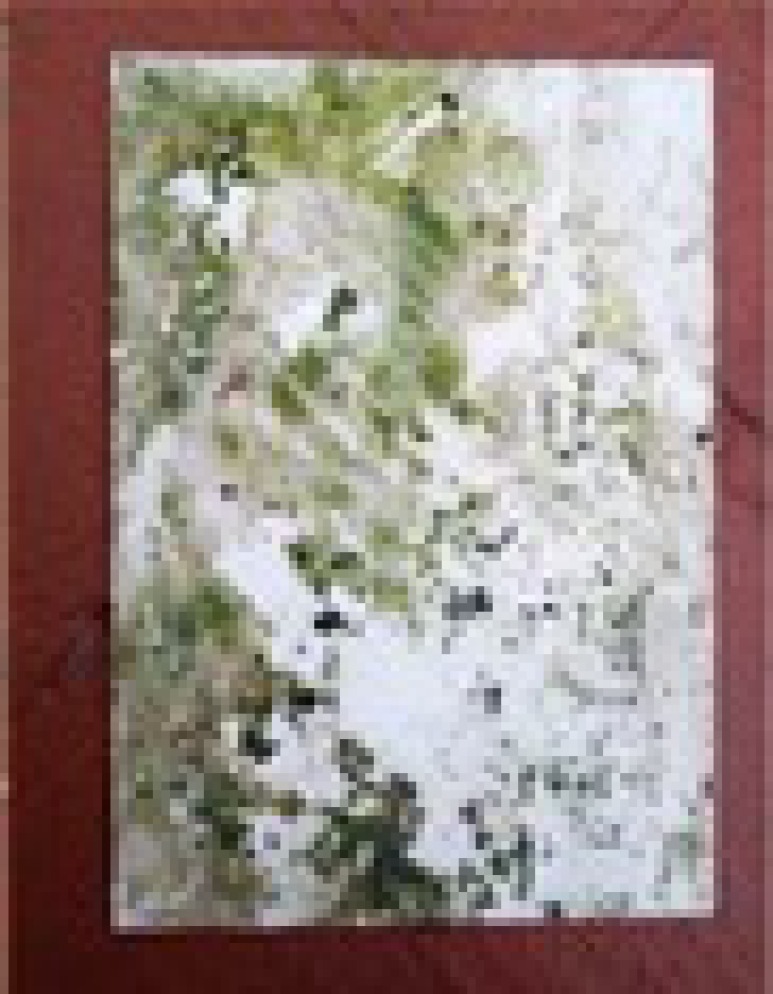
Resistant	High—did not present significant changes, and color didn't fade.	Medium—presented color changes and buckling.	Very high—Ideal for outdoor use; withstands thermal changes well.	Very Low—Warps when absorbing water, and color changes significantly.	Low—it presents fungal growth.

At the outdoor exposure test, the resin‐based material showed the worst outdoor performance and was discarded, as it coiled up and flattened when exposed to water and sunlight. PETG showed fungal growth in the fourth month leading to its rejection. PP, ASA, and PLA performed well, showing no deformation or buckling. PLA's performance was particularly noteworthy; due to its biodegradable nature, decomposition was expected but did not occur during the experiment. Therefore, these last three options were evaluated in test 2.

Table 2 presents the results of the printability and dimensional stability test taking as a reference the top view of the upper piece of the camera mount to evaluate finishing quality, and its side view to evaluate dimensional stability.

**TABLE 2:** Results of the printability and dimensional stability test. Comparison of PLA (Poly Lactic Acid), PP (Polypropylene), and ASA (Acrylonitrile Styrene Acrylate) evaluating printability and dimensional stability of a camera mount piece prototype.

Material/Criteria	PLA (Poly Lactic Acid)	PP (Polypropylene)	ASA (Acrylonitrile Styrene Acrylate)
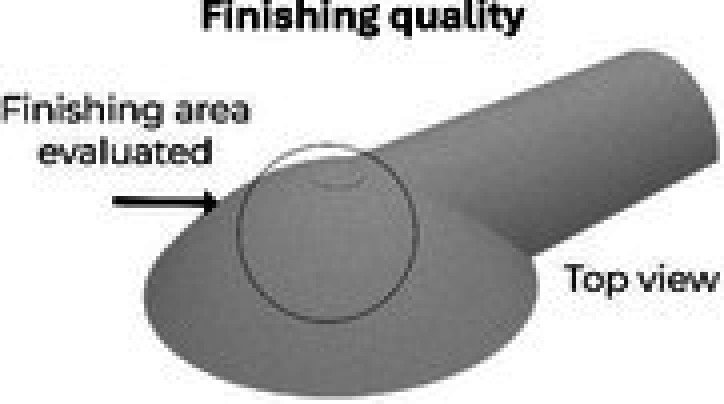	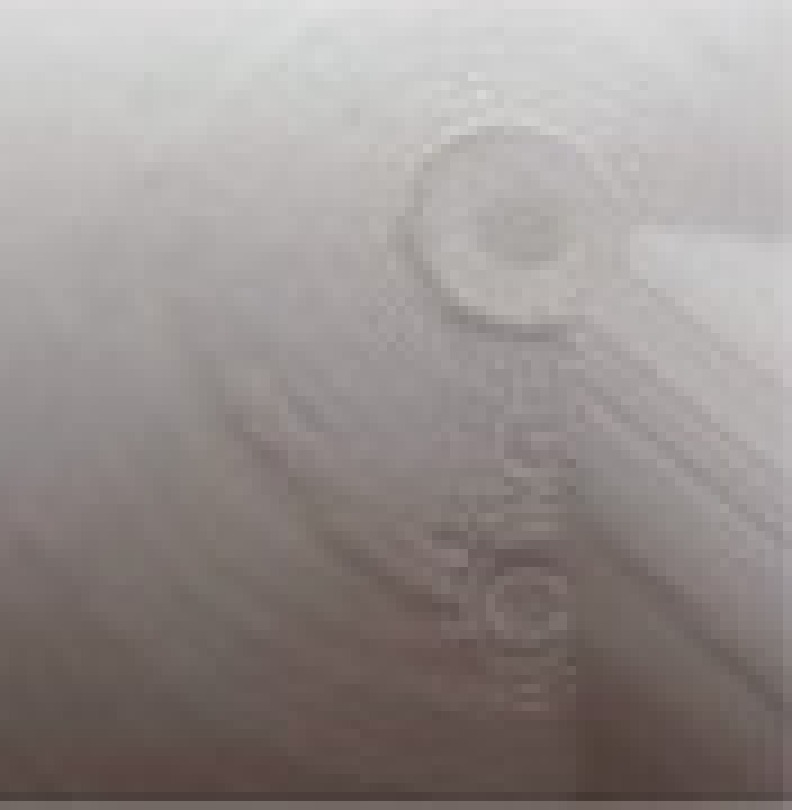	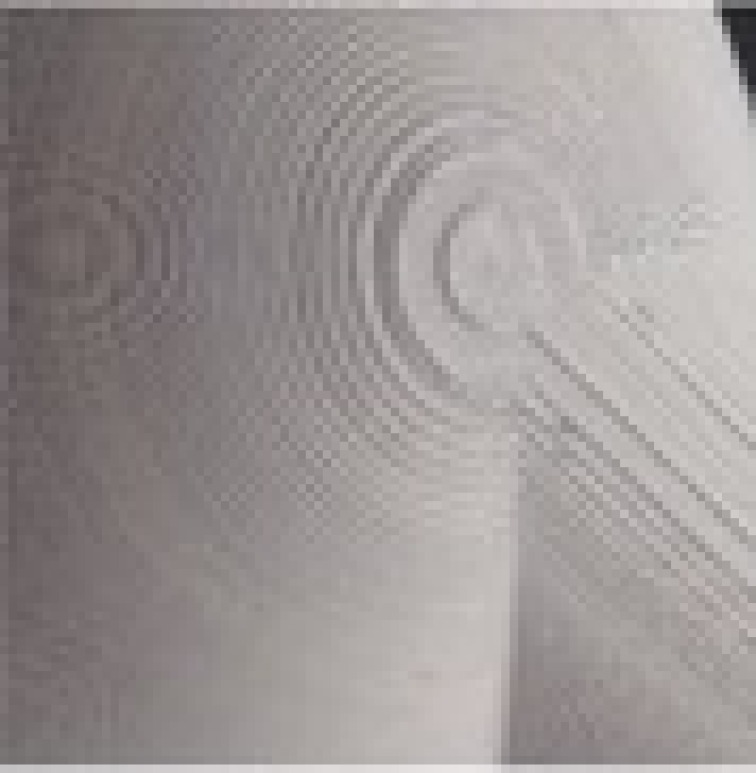	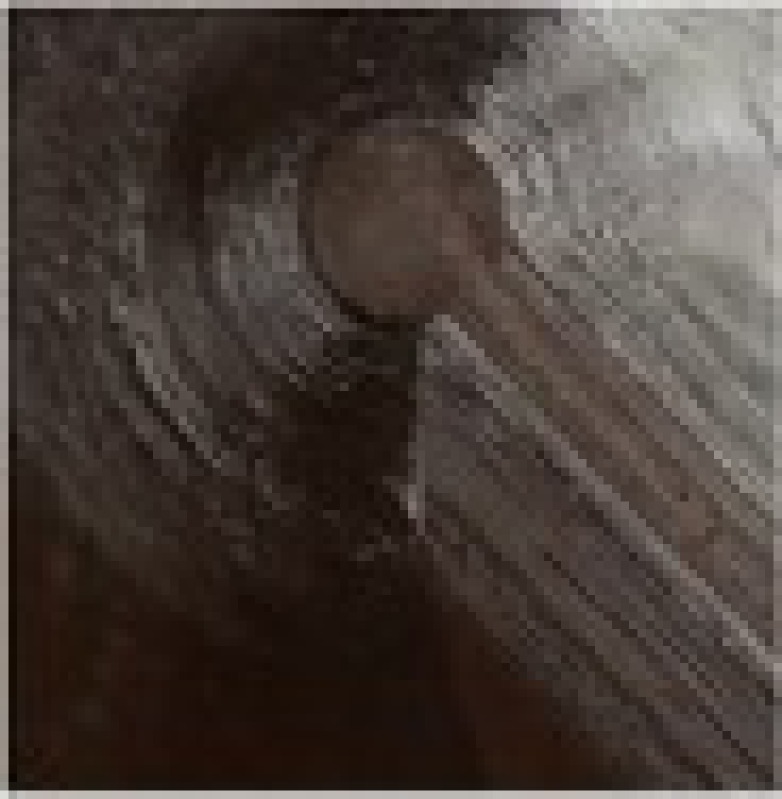
Observations	Easy to print, high quality achieved	Difficult to print. Presented poor bed adhesion, which causes severe deformation	Moderately challenging to print, but it offers good finishing quality. Time‐consuming and can present poor bed adhesion
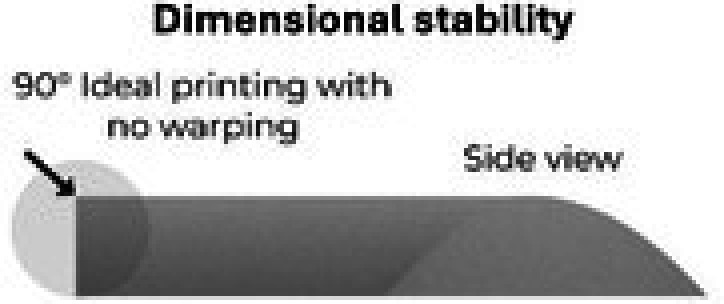	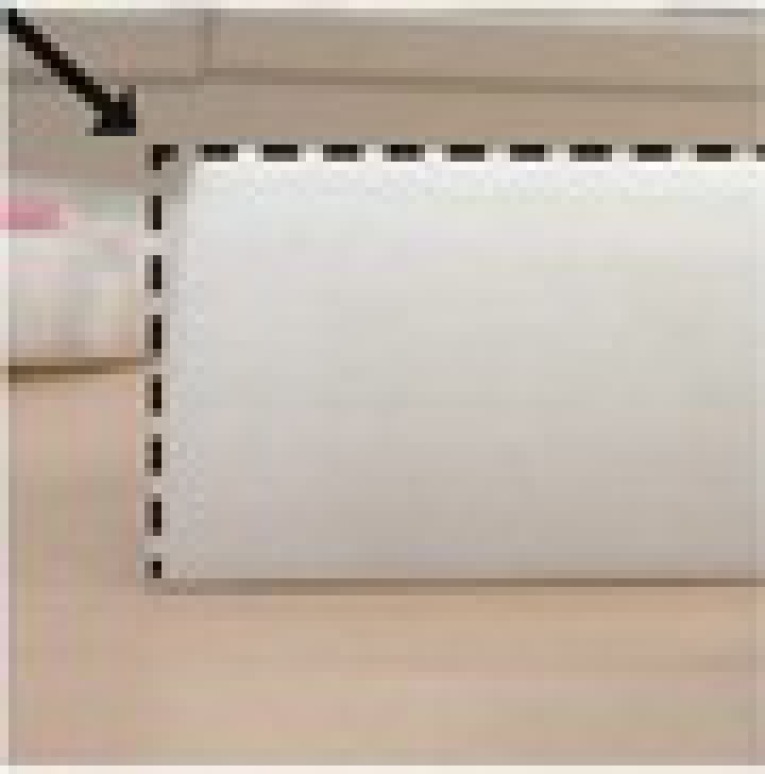	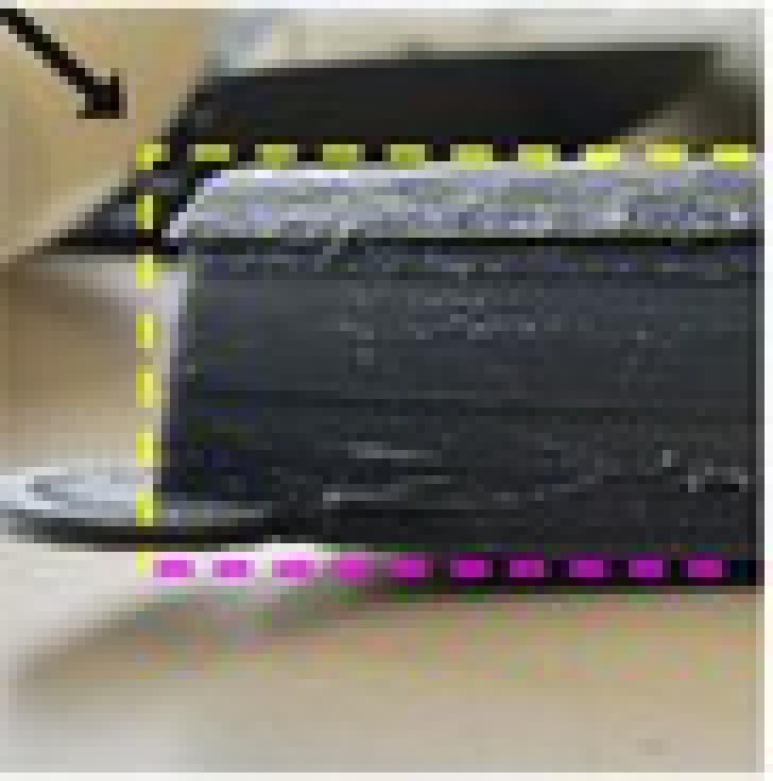	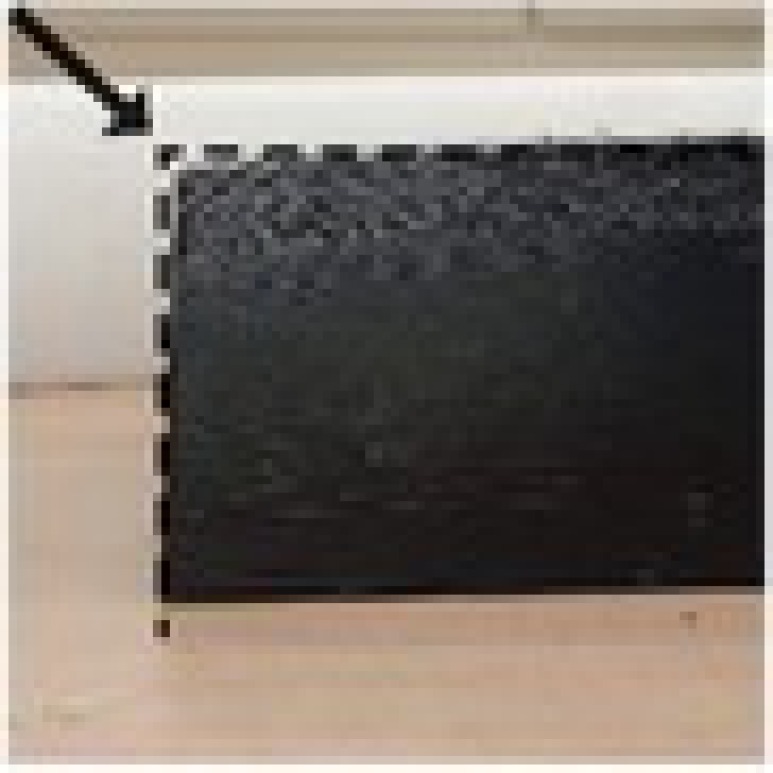
Observations	High–present dimensional changes at X axis	Warps significantly; therefore, some dimensions were altered	High dimensions correspond to the 3d model

This second test showed similar results concerning the printing quality of the three materials; however, PLA and ASA presented better dimensional stability than PP, which was challenging to print and showed some deformation due to warping. Its flexibility resulted in geometric instability, making it unsuitable for the mounting and housing elements application. PLA and ASA offered good performance in the printing process, although ASA required careful handling to avoid warping. Despite the excellent printing properties of PLA, it was discarded due to concerns about potential long‐term biodegradation. Therefore, the camera and the radar mount final pieces were printed in ASA. This filament maintained its shape without structural damage and adequately withstood thermal changes during testing.

Concerning coatings test, Table 3 summarizes the results of the salt spray chamber test described in Section 2.3.3, including sample photographs pre‐ and post‐test, a classification of feasibility, and a technical conclusion which considered ease of application, technology required, cost, and curing time.

**TABLE 3:** Results of salt spray chamber coatings tests. Summary of material performance after 100 h exposure to a 5% NaCl salt spray environment at 35°C and 90% relative humidity, conducted according to ASTM B117‐25. The table compares the corrosion behavior of uncoated and protected cold‐rolled steel sheets, as well as the performance of different paint and film‐based camouflage alternatives applied to metal and plastic samples. Feasibility classification considers corrosion resistance, coating adhesion, surface degradation, ease of application, cost, and curing requirements.

Salt spray chamber corrosion test results						
Aim	Sample	Pre‐test	Post test	Result	Considerations	Feasibility
No coat	Cold‐rolled uncoated	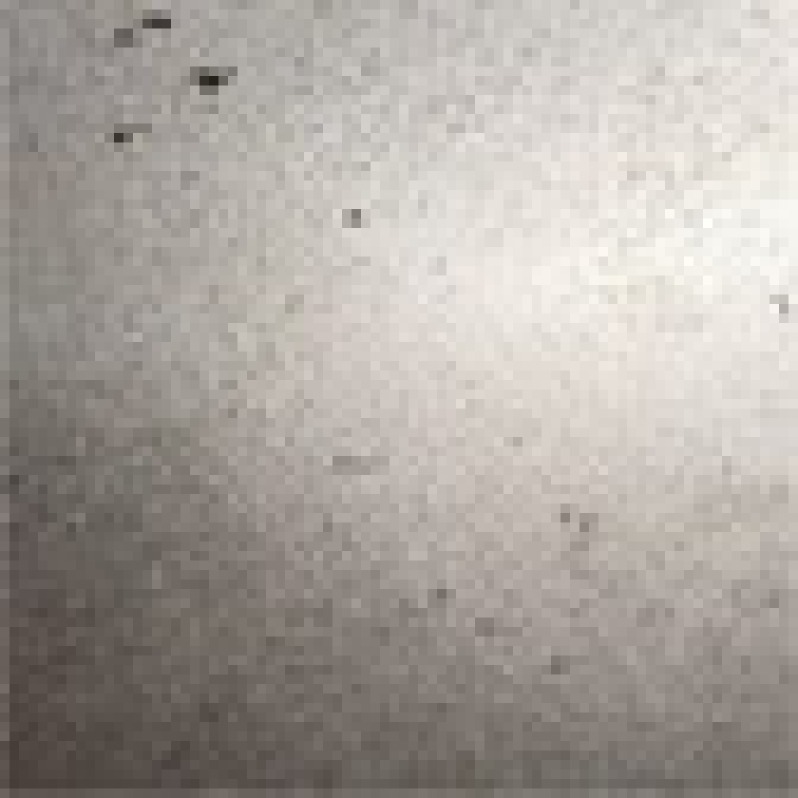	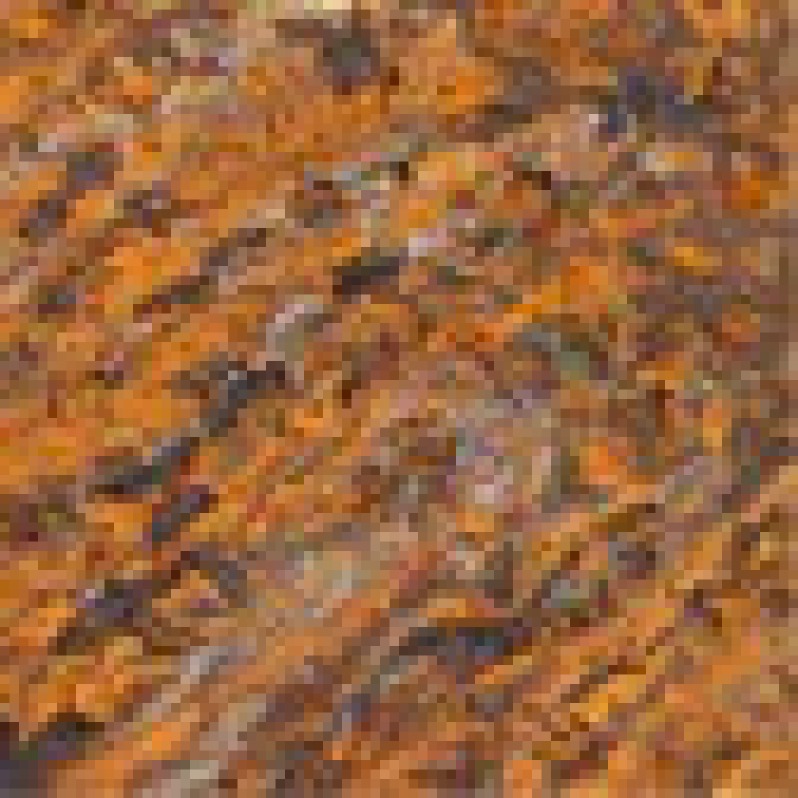	Severe corrosion, red rust marks	Not suitable for humid or saline environments.	❌ Very low
Corrosion inhibitor	Cold‐rolled galvanized	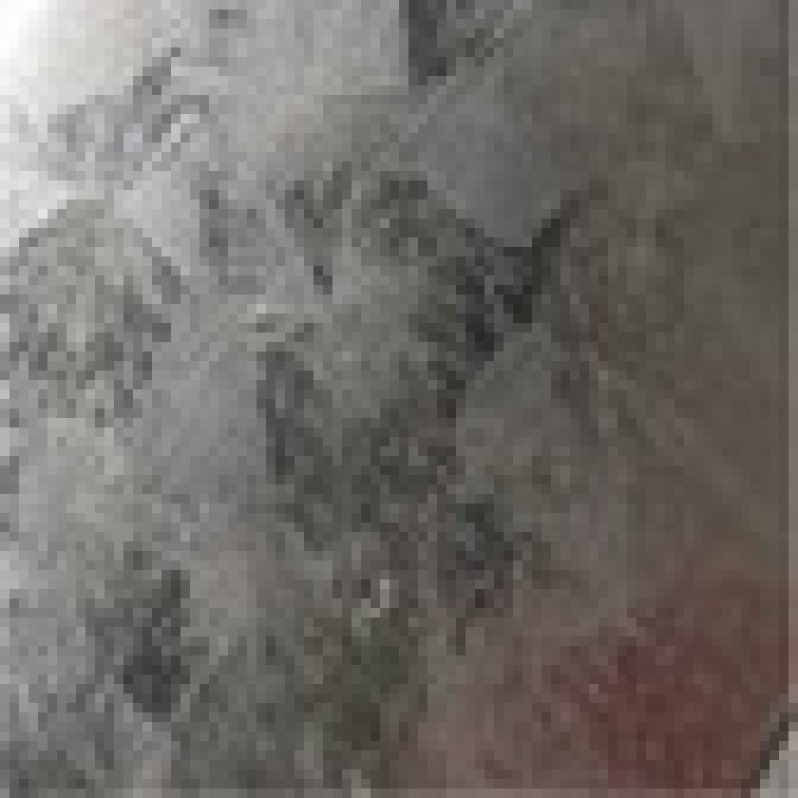	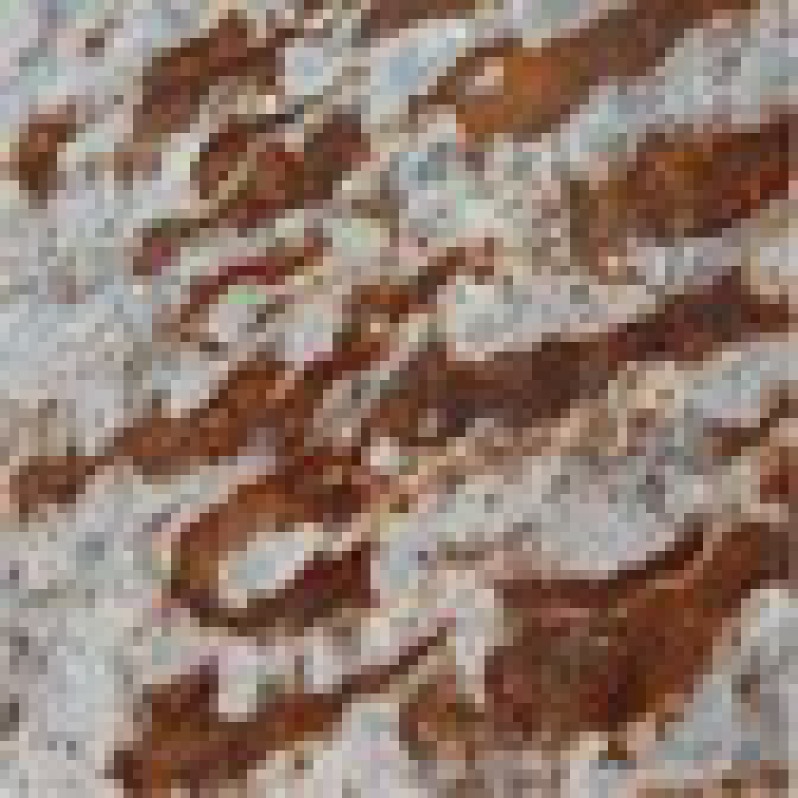	White rust and red marks	Less corrosion than cold‐rolled uncoated, although it presents oxide marks.	❌ Low
	Anticorrosion paint	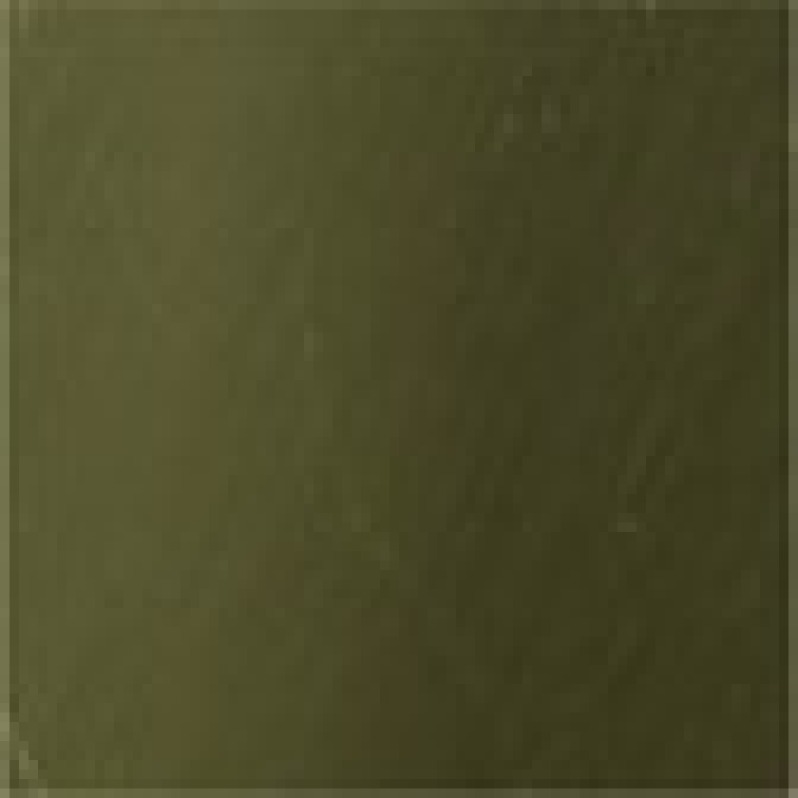	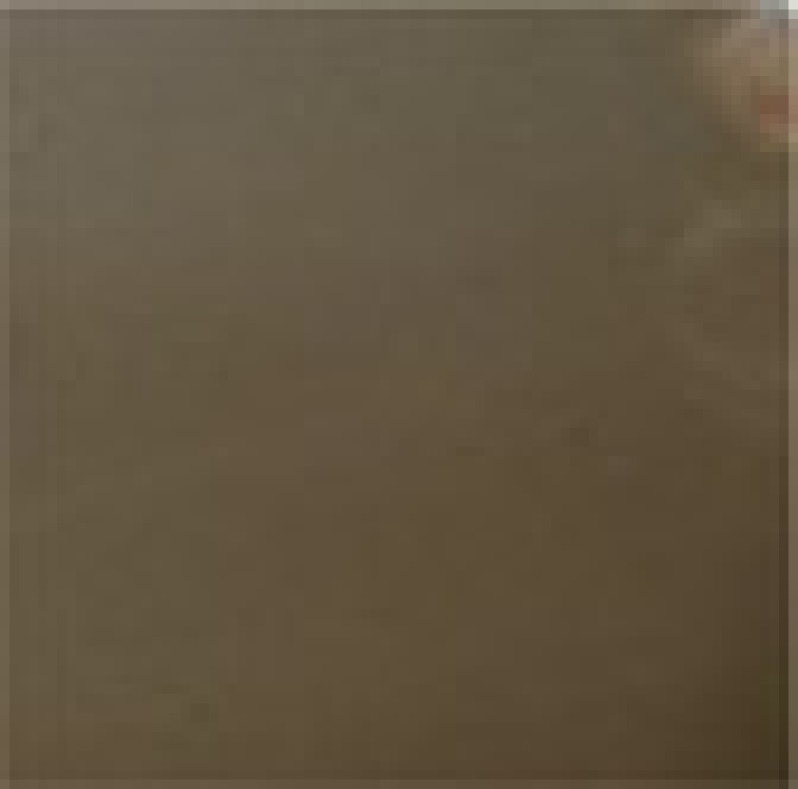	Slight spot of corrosion	Good barrier, but some punctual wear present.	✅ Good
Camouflage layer option 1: paint	Electrostatic paint	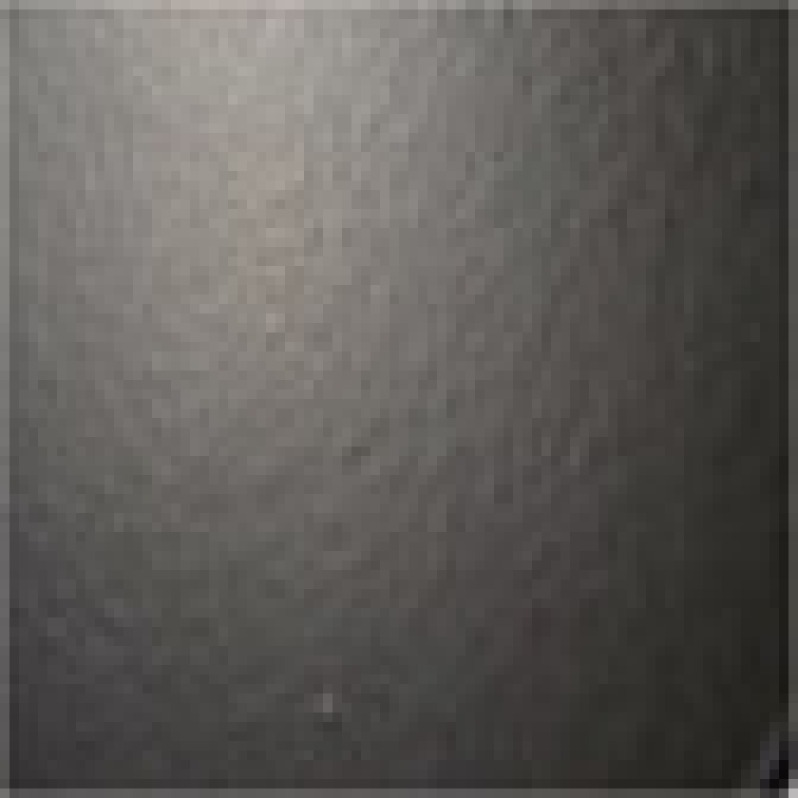	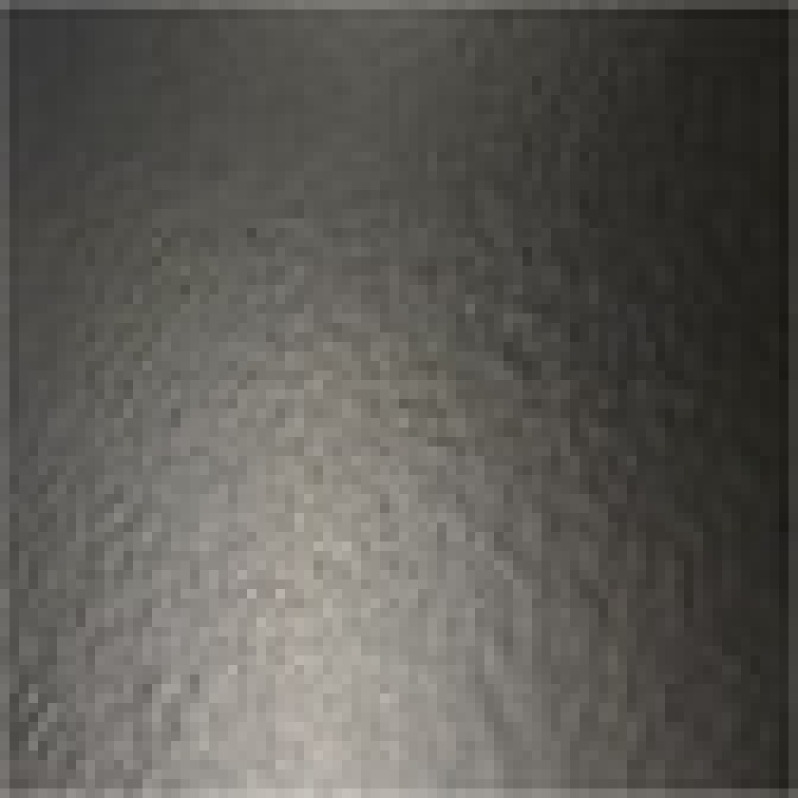	No visible changes	Very high resistance, although it needs special painting equipment and an oven to cure. It has very limited color options.	✅✅ Excellent
	Epoxy paint	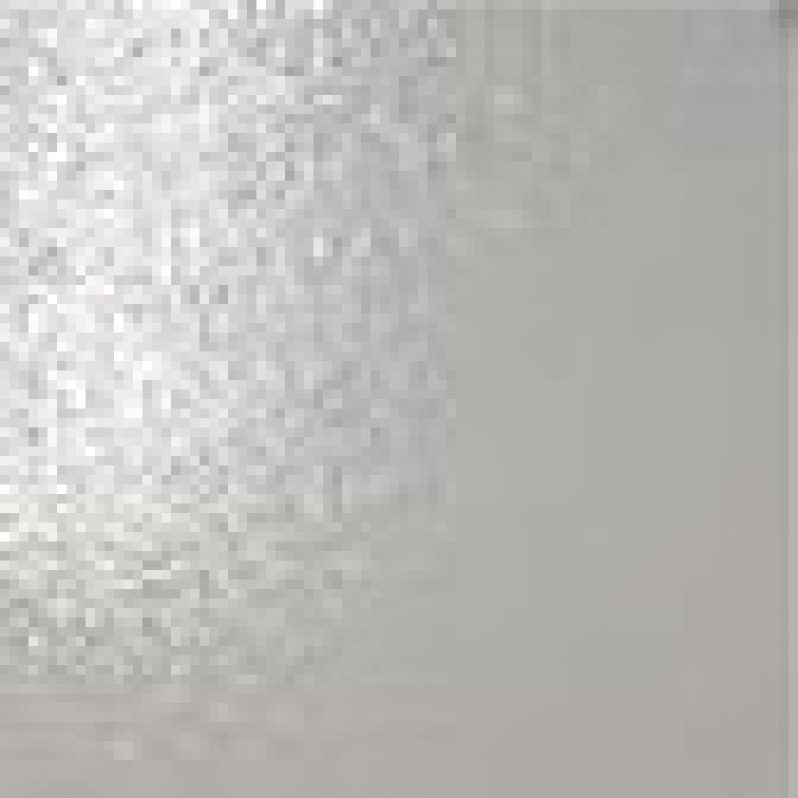	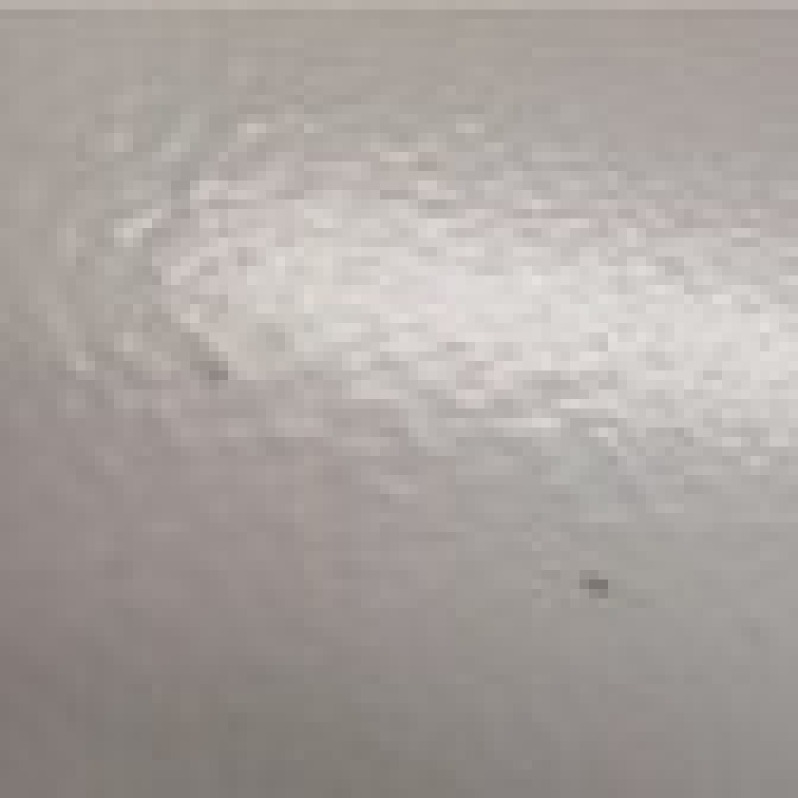	Loss of bright and small points of rust	Adhesion is not so good. Paint easily falls off when scratched. It requires a long time to dry.	⚠ Medium
	Lacquer paint	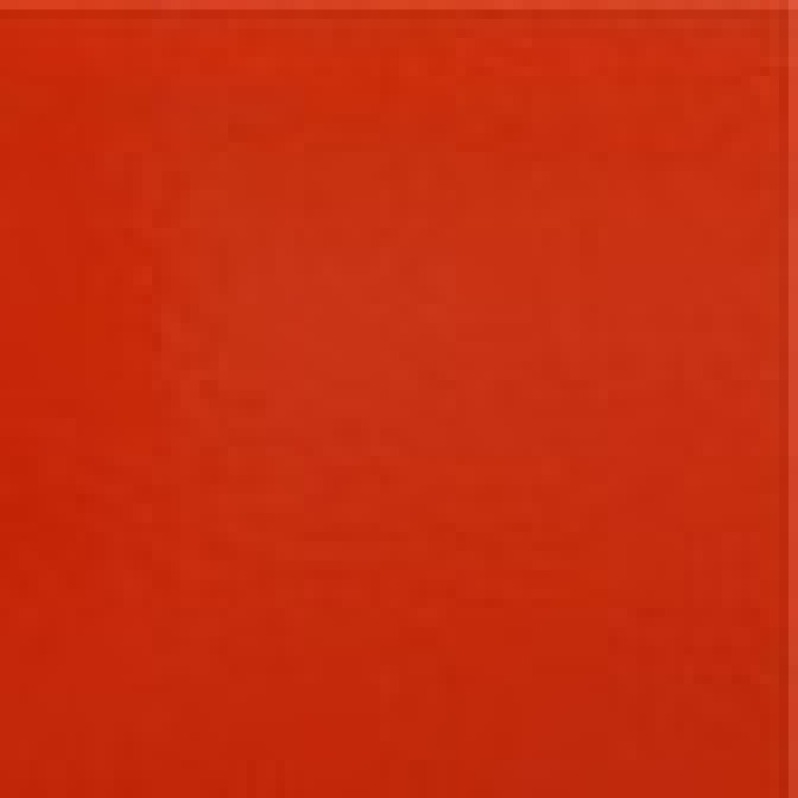	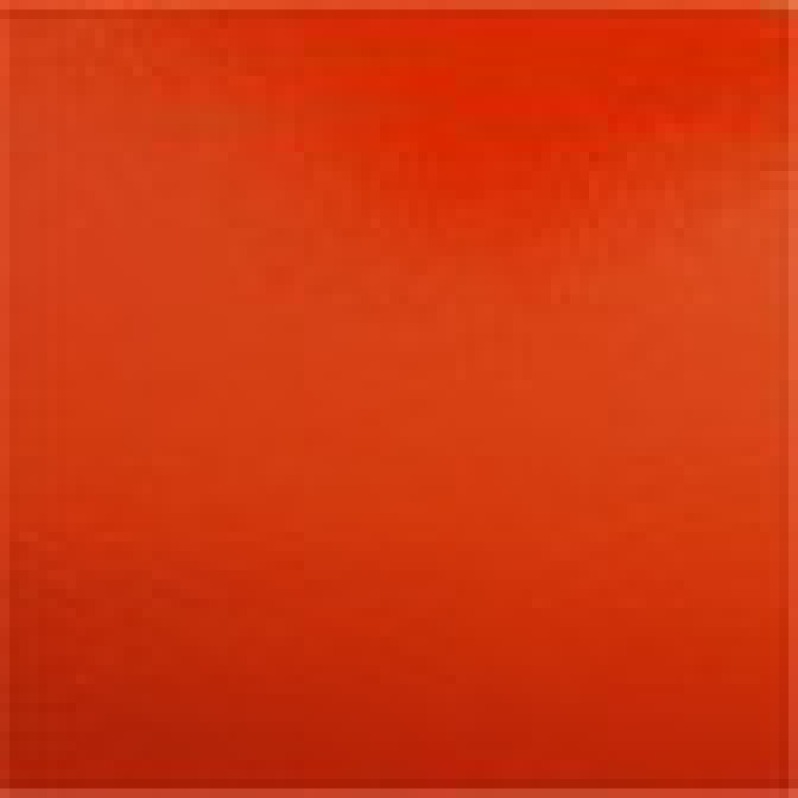	No visible damage	Good barrier, although it requires a long time to dry.	✅ Good
	Polyester paint	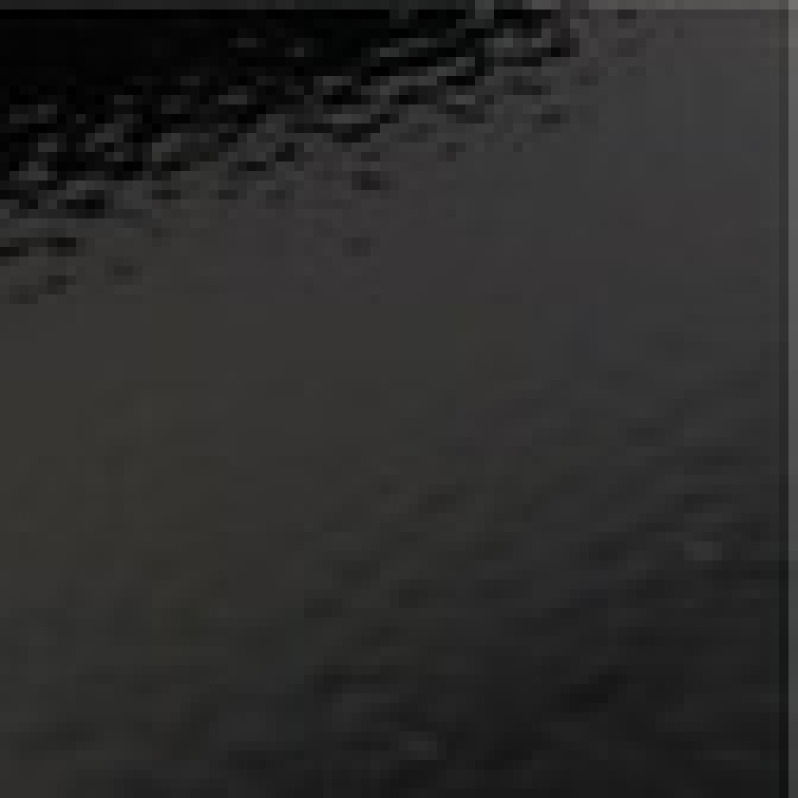	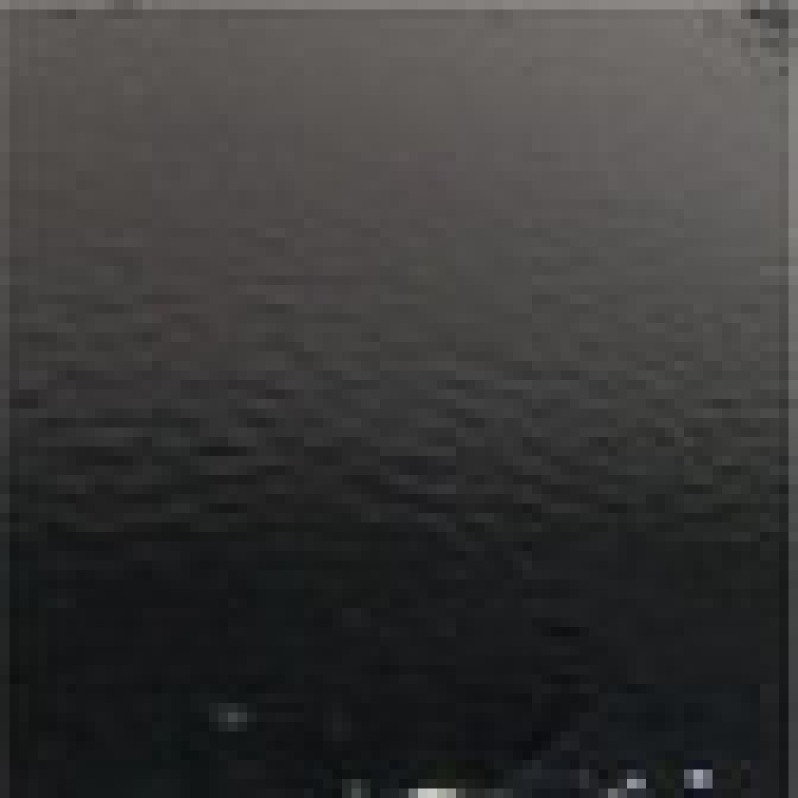	No visible damage	Very high resistance. It has a wide range of colors. Usually used for car painting.	✅✅ Excellent
	Polyurethane paint	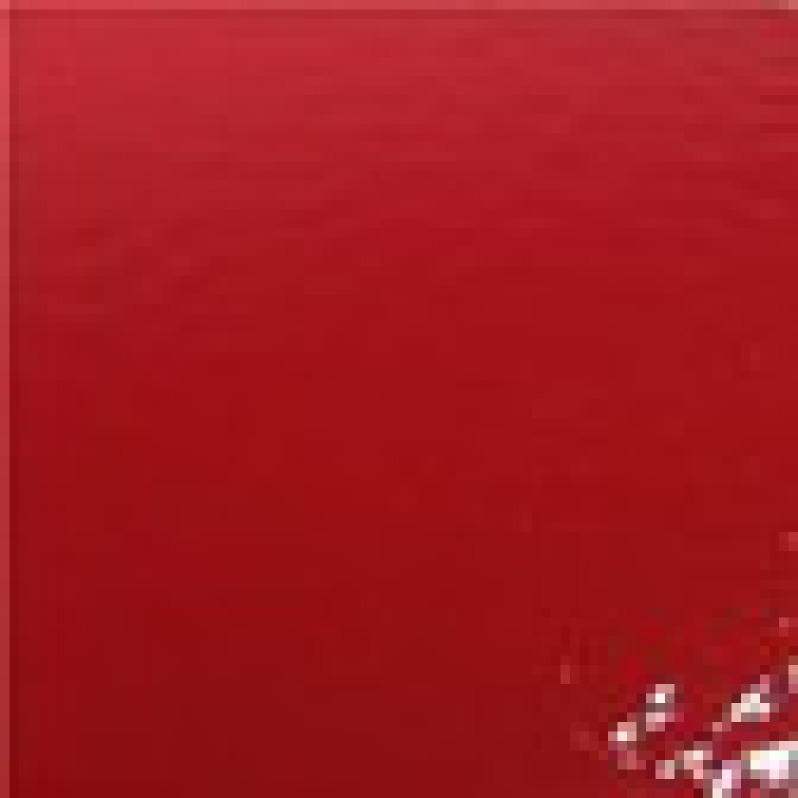	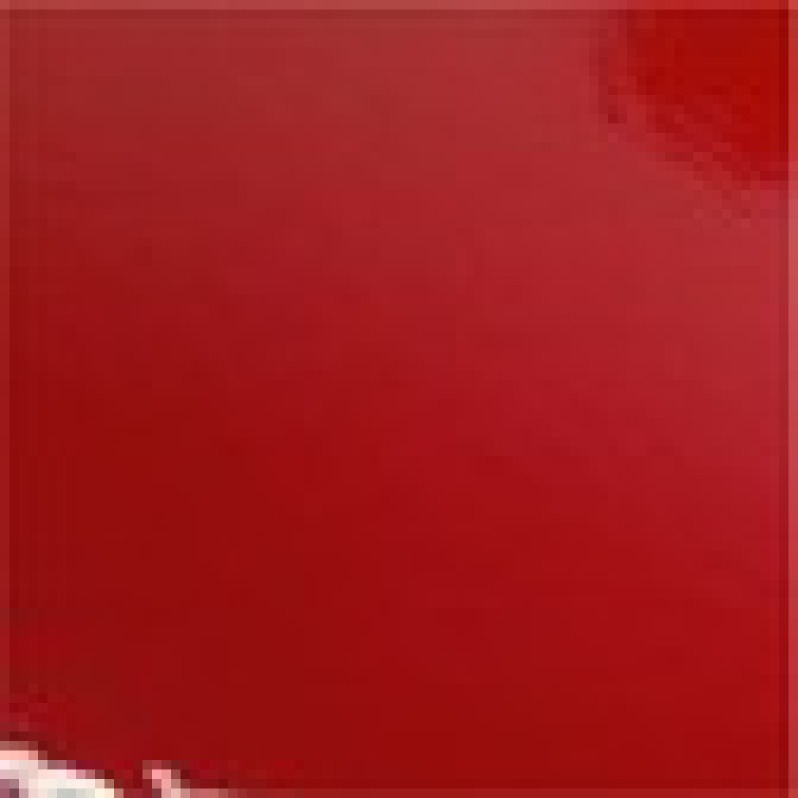	No visible damage	Good barrier, but the application process is difficult. The results vary depending on the environmental conditions on the day of application.	✅ Good
	Enamel paint	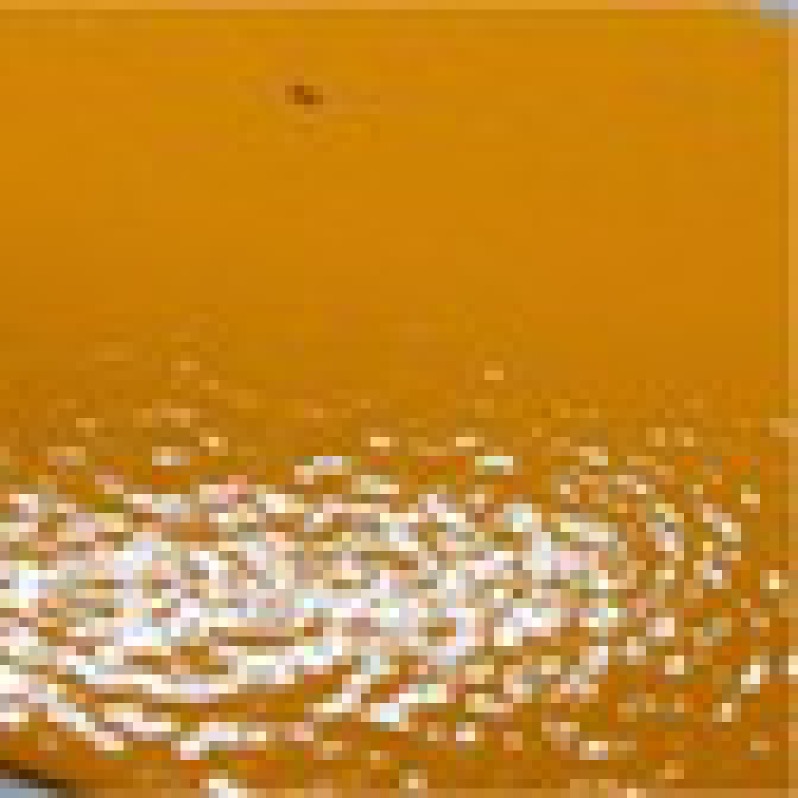	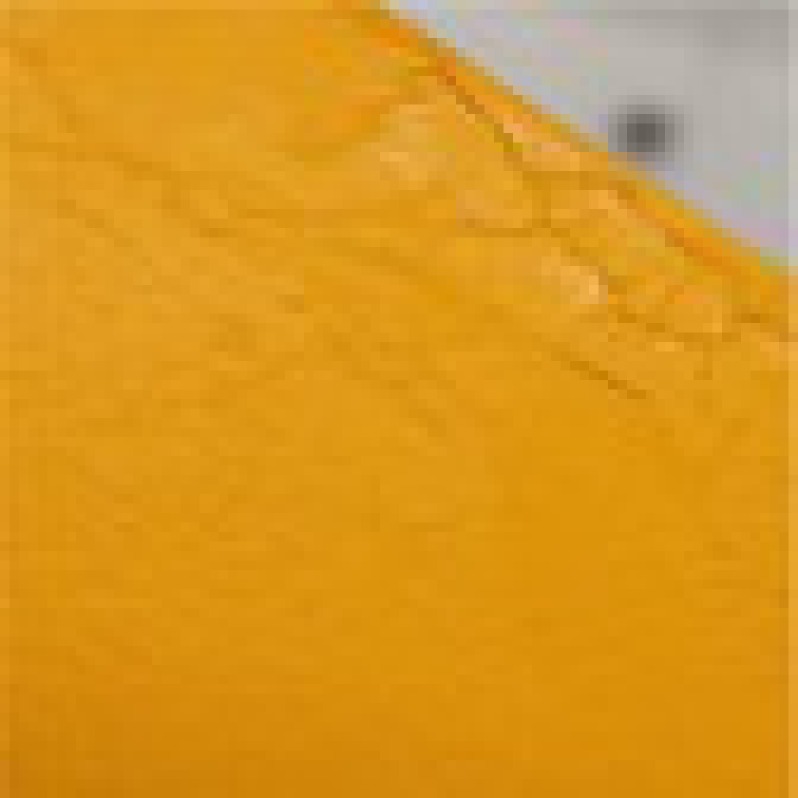	Cracking and loss of gloss.	Adhesion is not good, and the drying process is time‐consuming.	❌ Low
	Graffiti paint	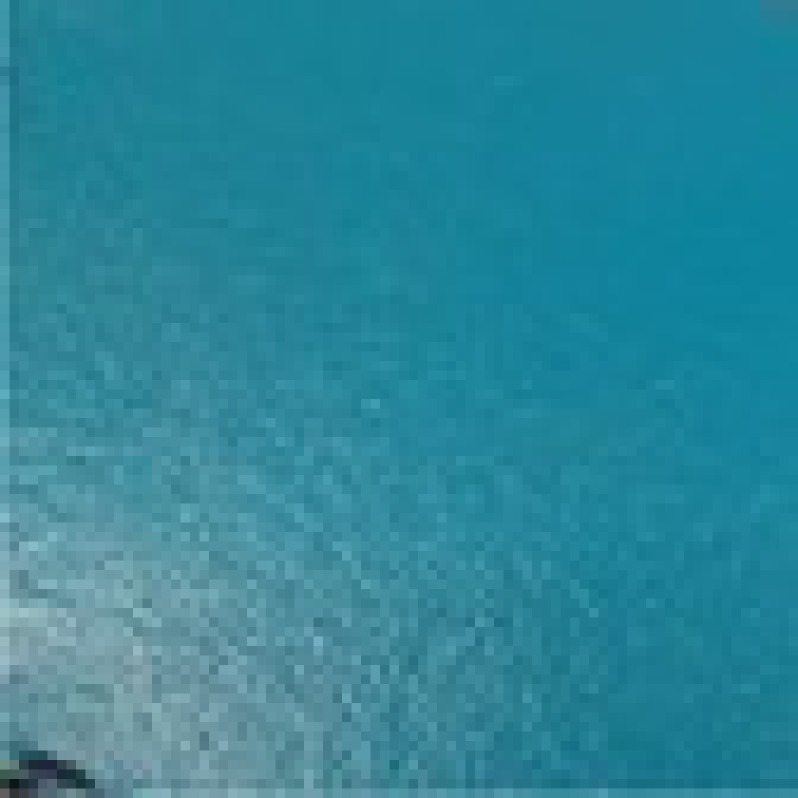	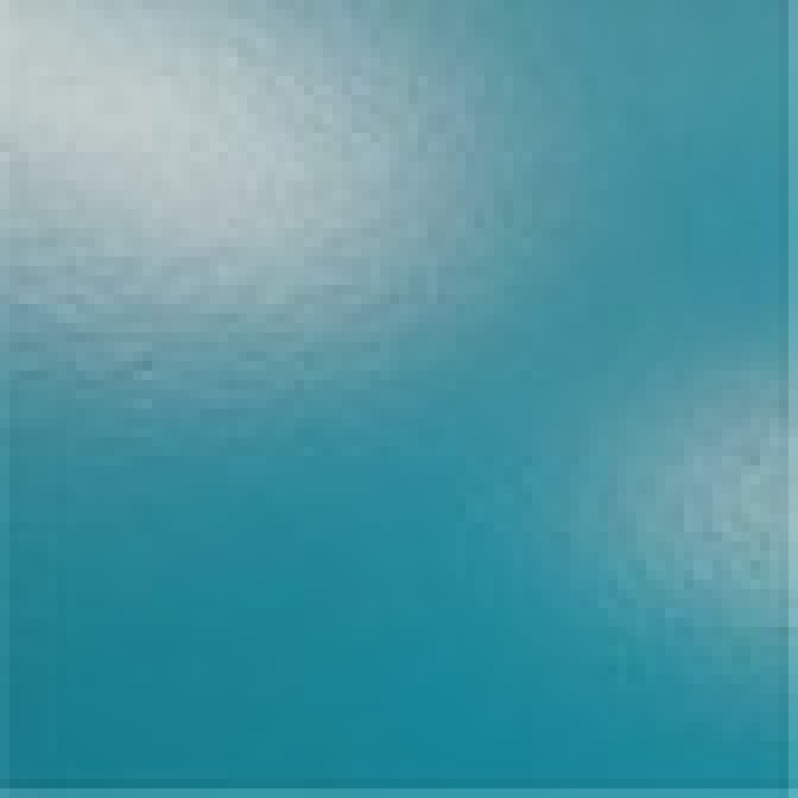	No visible changes	Good resistance and an easy application process. Commercially available in a wide range of colors.	✅✅ Excellent
Camouflage layer option 2: films	Car wrapping vinyl	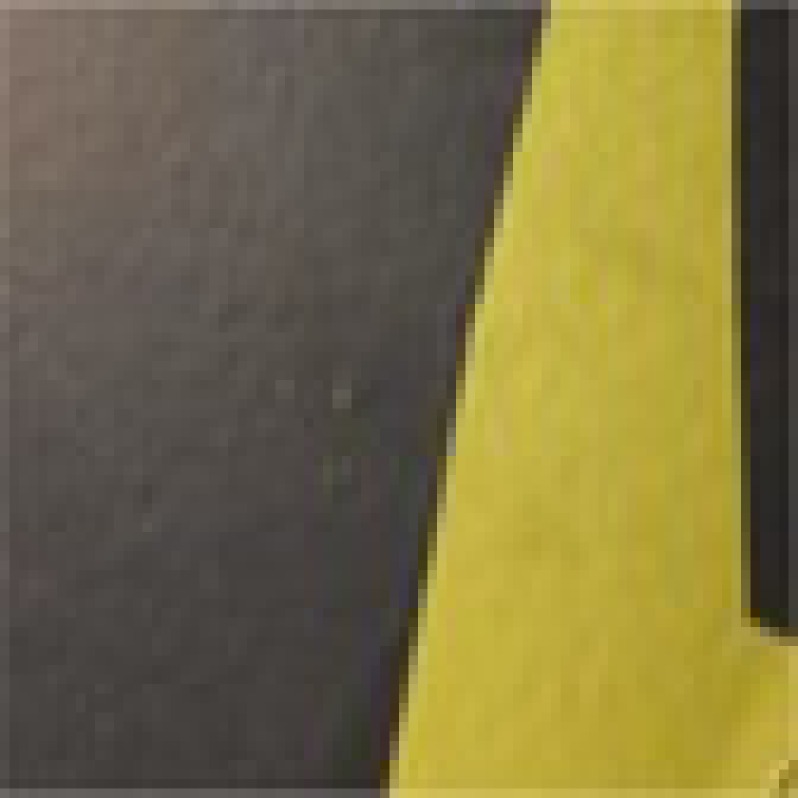	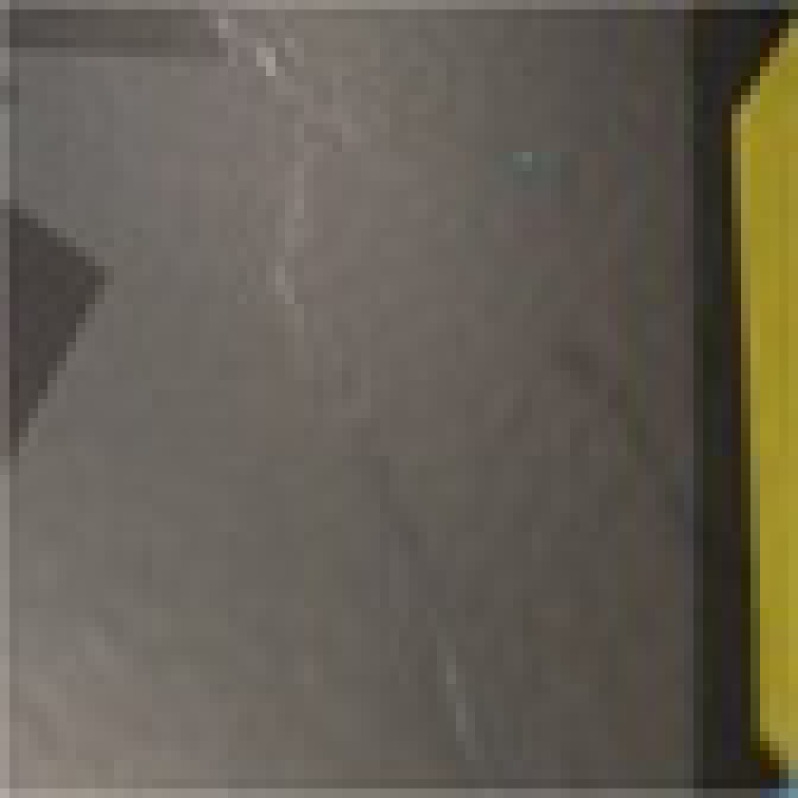	Partial detachment at the edges	Difficult application process for complex geometries. Very high cost. It offers a non‐permanent option, as it is removable.	⚠ Medium
	Water transfer Printing film	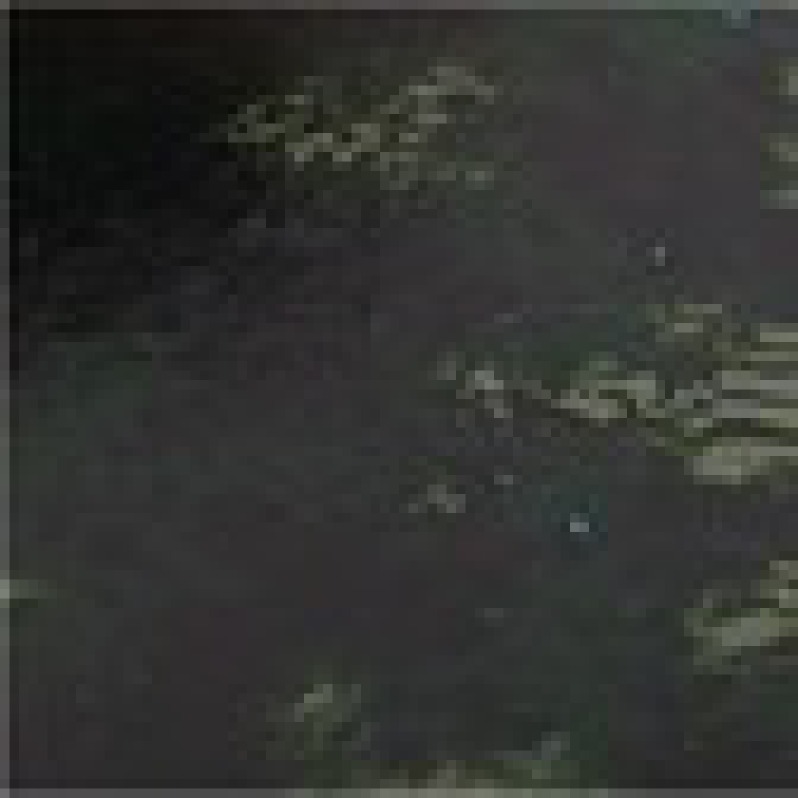	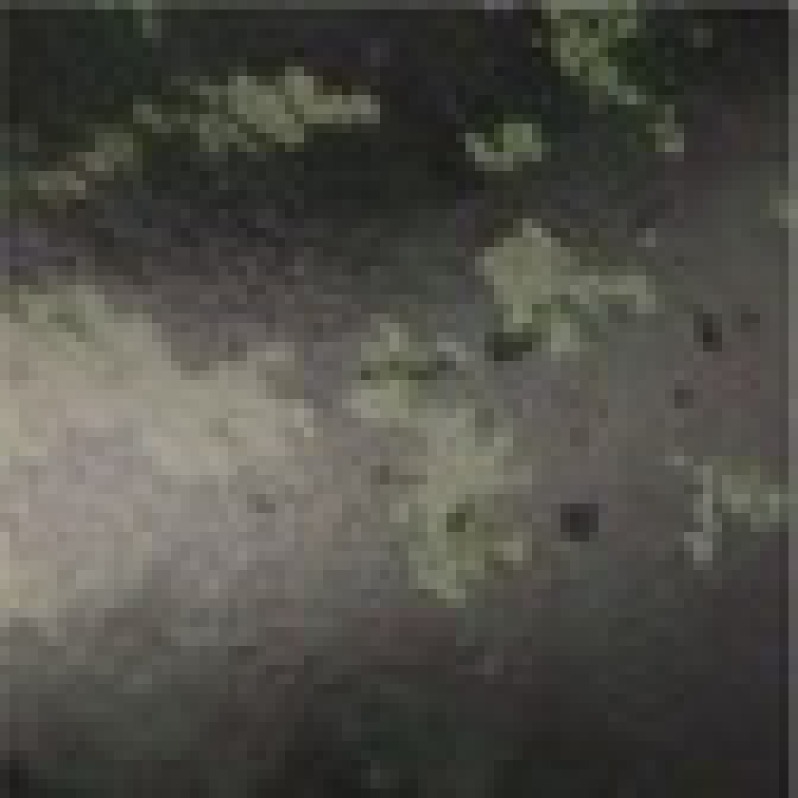	Loss of gloss	Simplifies the camouflage process. High water consuming	⚠ Medium
Coatings for plastics	Water transfer Printing film on plastic sheet	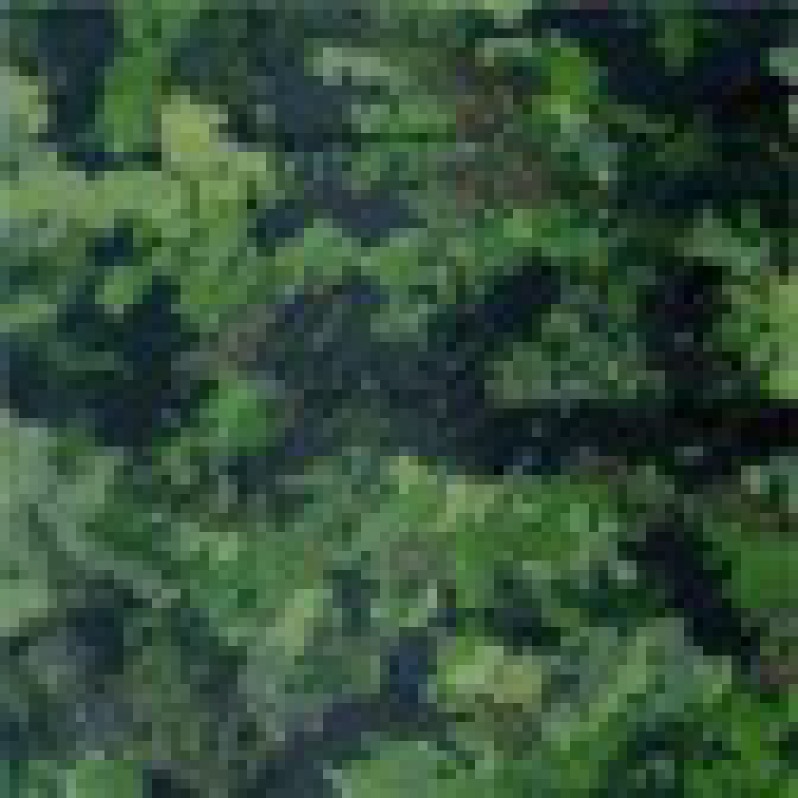	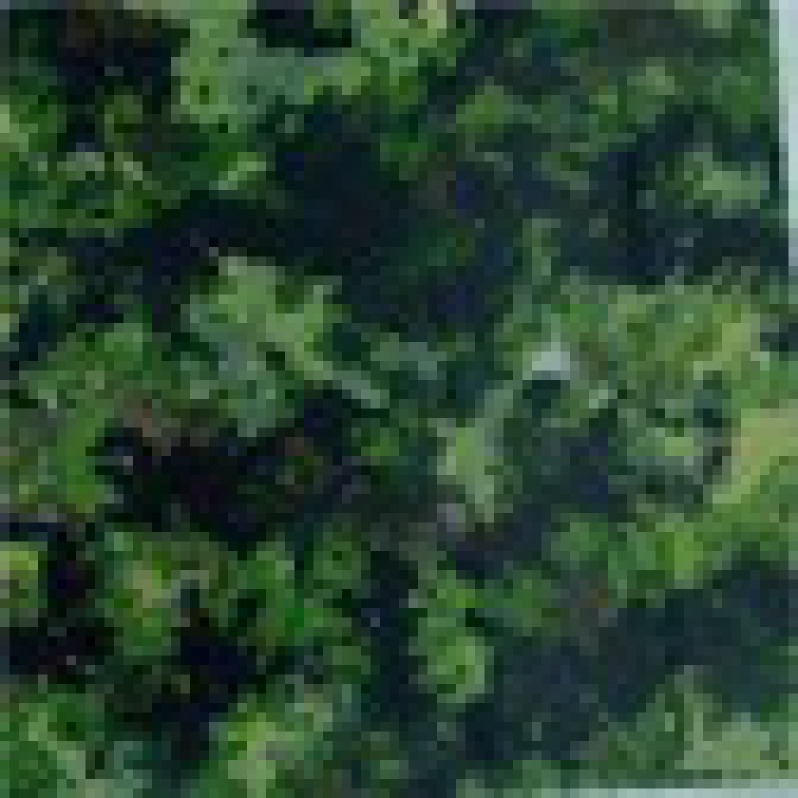	No visible change	Plastic required two layers of film to achieve good coverage. High water consuming.	✅ Good
	Water transfer Printing film on cable protection	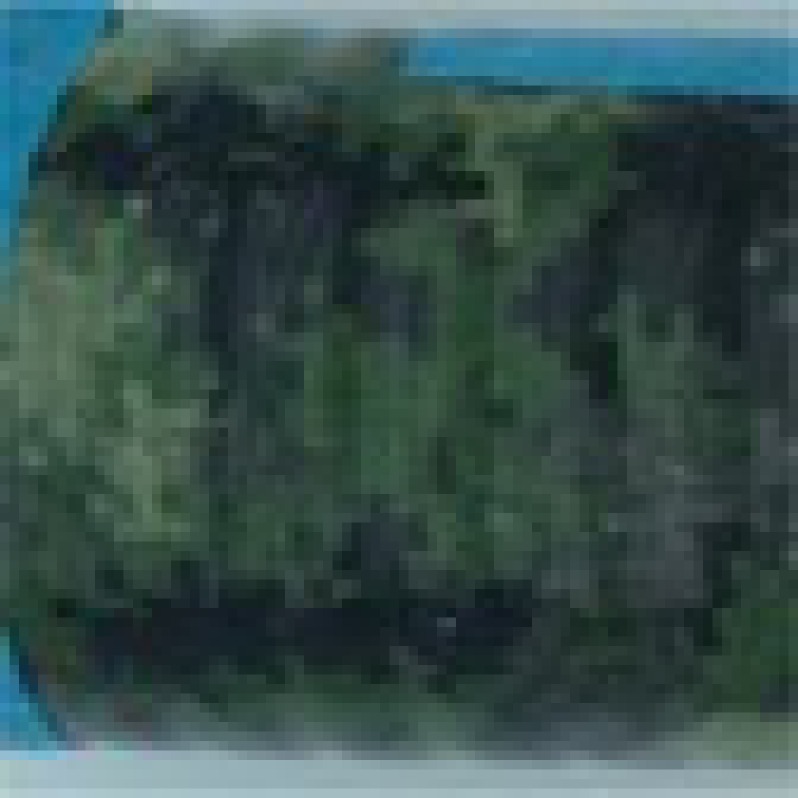	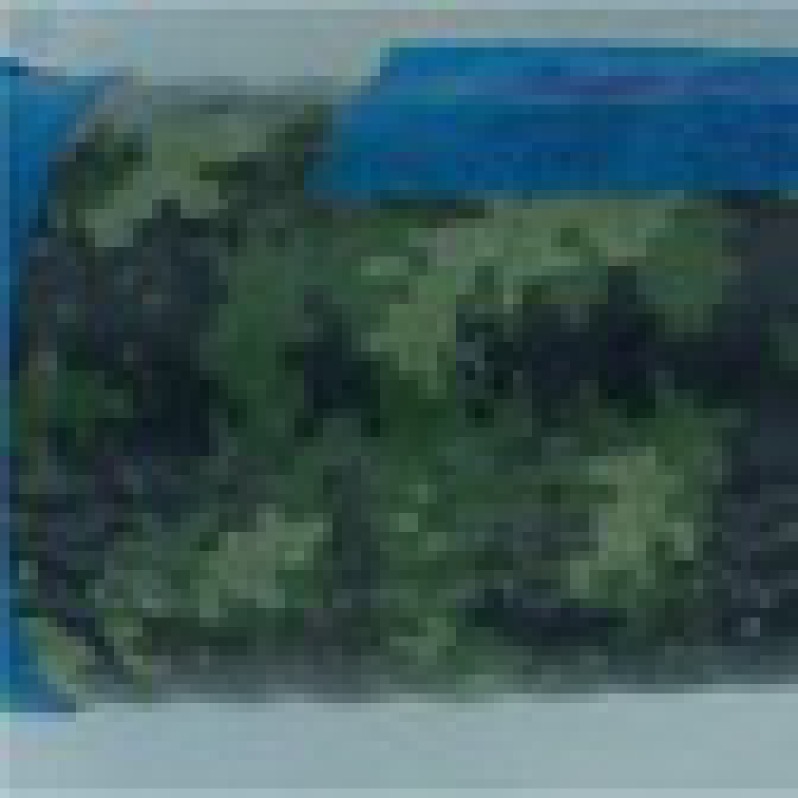	No visible change		✅ Good
	Graffiti paint	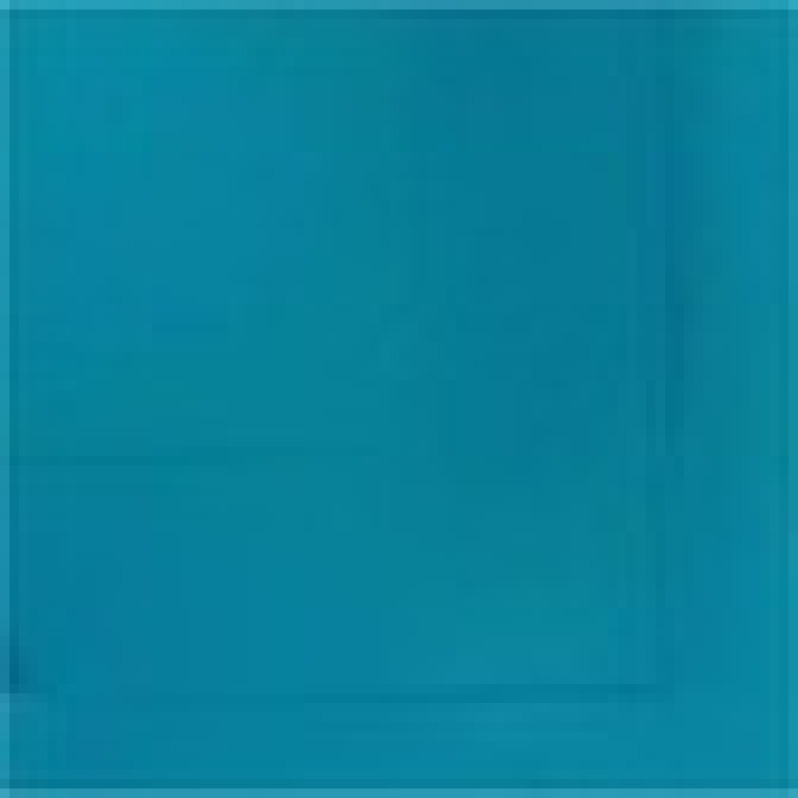	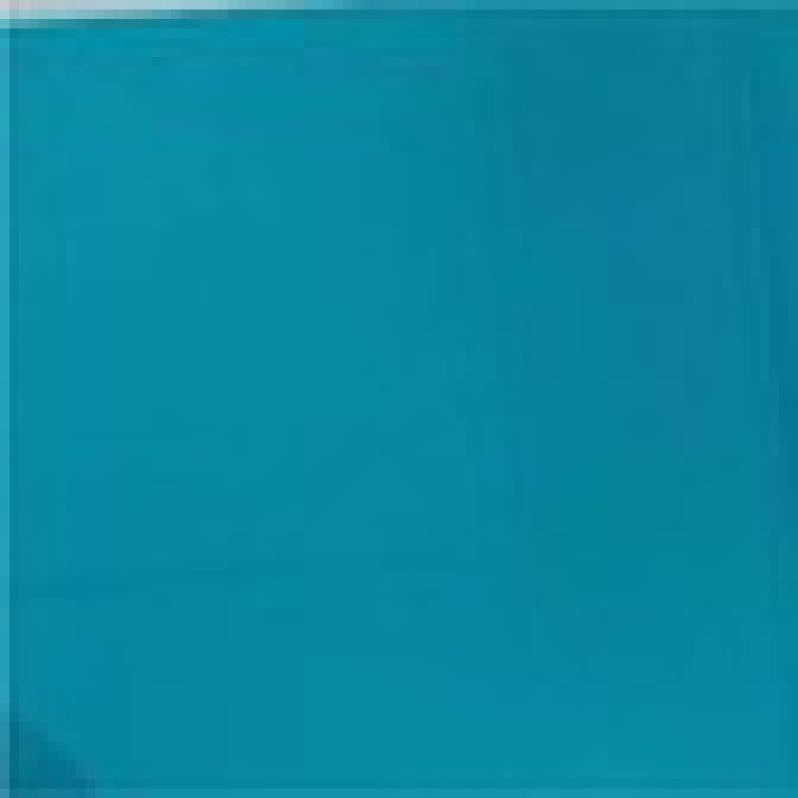	No visible change	Good adhesion on plastic. Additionally, it offers a matte finish.	✅ Good

Concerning the metals test, the uncoated sheet exposed in the salt spray chamber showed severe corrosion, establishing a reference point to compare the corrosion inhibitor options: the galvanized coating and the anti‐corrosive paint. Both provided a good level of protection; however, the galvanized sheet exhibited significant white and red rust marks, whereas the anti‐corrosive paint only showed localized wear. Thus, anti‐corrosive paint proved to be the most effective option for the humid conditions of the Amazon rainforest.

Regarding the paint alternatives, polyester, polyurethane, lacquer, and graffiti paint performed well, showing no visible changes after the test, with no evidence of cracking, peeling, or loss of gloss. In contrast, epoxy paint presented adhesion deficiencies, while enamel showed cracking, reducing its viability. For film‐based alternatives, the car‐wrapping vinyl sticker and water‐transfer printing film also produced acceptable results, with only partial peeling or loss of gloss observed.

According to the test results, several alternatives for both options (paints and film‐based) could be selected. However, graffiti paint was selected due to its wide color palette, its low cost, and its ease of application since it does not require additional application equipment. It performed optimally on both plastics and metals, which simplifies logistics.

**FIGURE 5:**
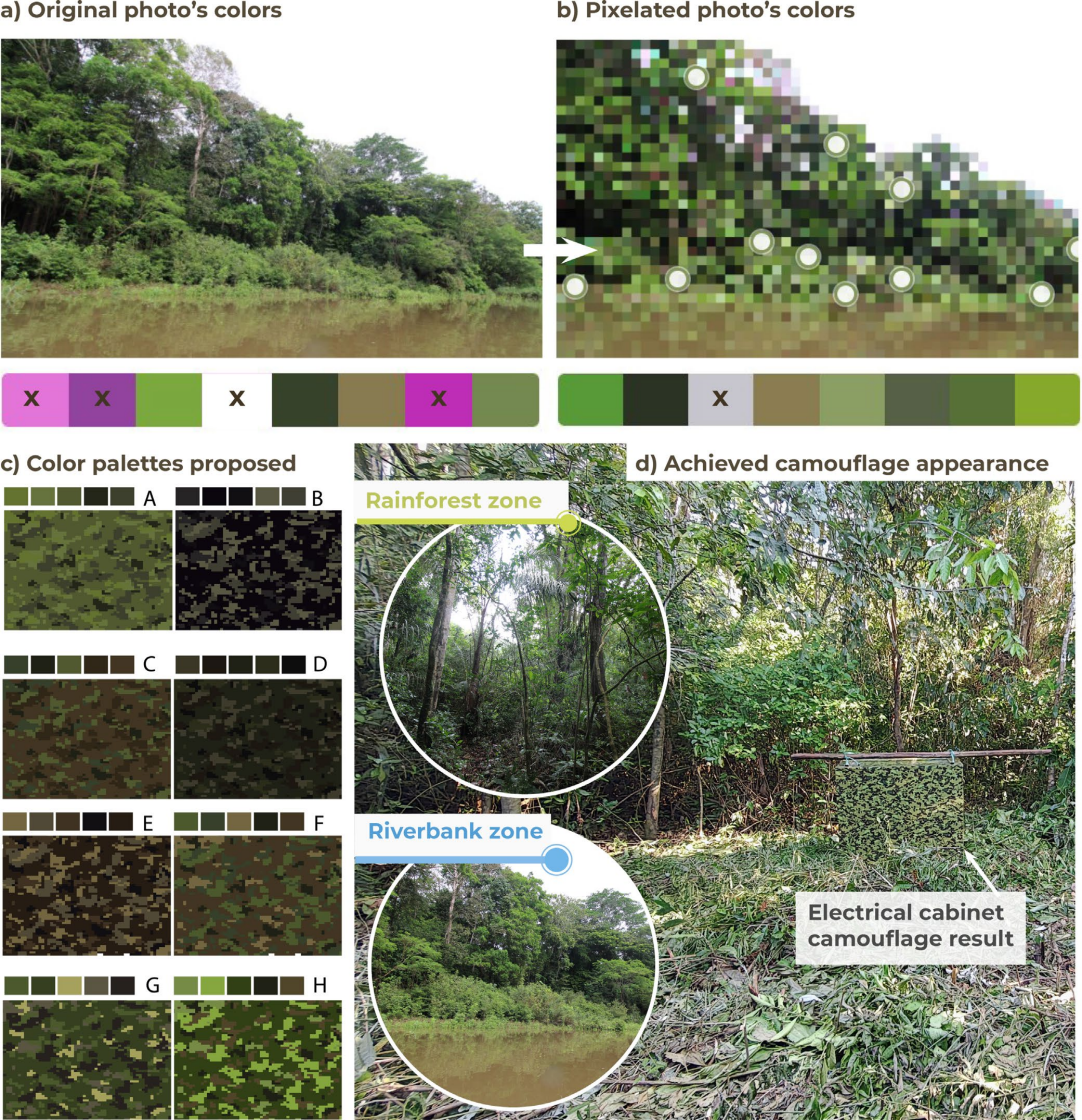
Environmental reference photo and derived camouflage color palettes. (a) Original environmental photo from the Colombian Amazon rainforest used as reference for color extraction. (b) Pixelated version of the same photo used to simplify the color distribution and identify dominant tones. (c) Eight camouflage color palettes derived from the environmental photos, evaluated for visual coherence with the surrounding vegetation and soil elements. (d) Final camouflage appearance of the electrical cabinet installed at Amacayacu National Natural Park (ANNP), illustrating the visual integration of the mounting and housing elements within the environment.

Regarding color selection, Figure 5a shows an environmental reference photo. Figure 5b presents the colors detected by a palette generator software of the original photo and its pixelated version, showing that without the pixelated improvement, the colors could include non‐coherent options such as pink. Several photos provided 15 color options that were mixed to generate the eight color palettes shown in Figure 5c.

The selected palette (H in Figure 5c) is shown applied to the electrical cabinet at ANNP, demonstrating visual integration within the surrounding environment (Figure 5d). When the colors were printed and tested visually from different distances, most of the palettes appeared almost monochromatic, enhancing visibility. This led to the selection of palette H because its brighter tones in smaller proportions improved the contrast and camouflaged the mounting and housing elements into the environment. Palette H contained the following Montana colors, listed in order of application along with their Pantone codes: Comarca Green (5747 U) as the base, followed by Green Infinity (5605 U), Chocolate Brown (412 U), Dharma Green (5535 U), and Euskadi Green (378 U).

### 3.3 Installation Results

**FIGURE 6:**
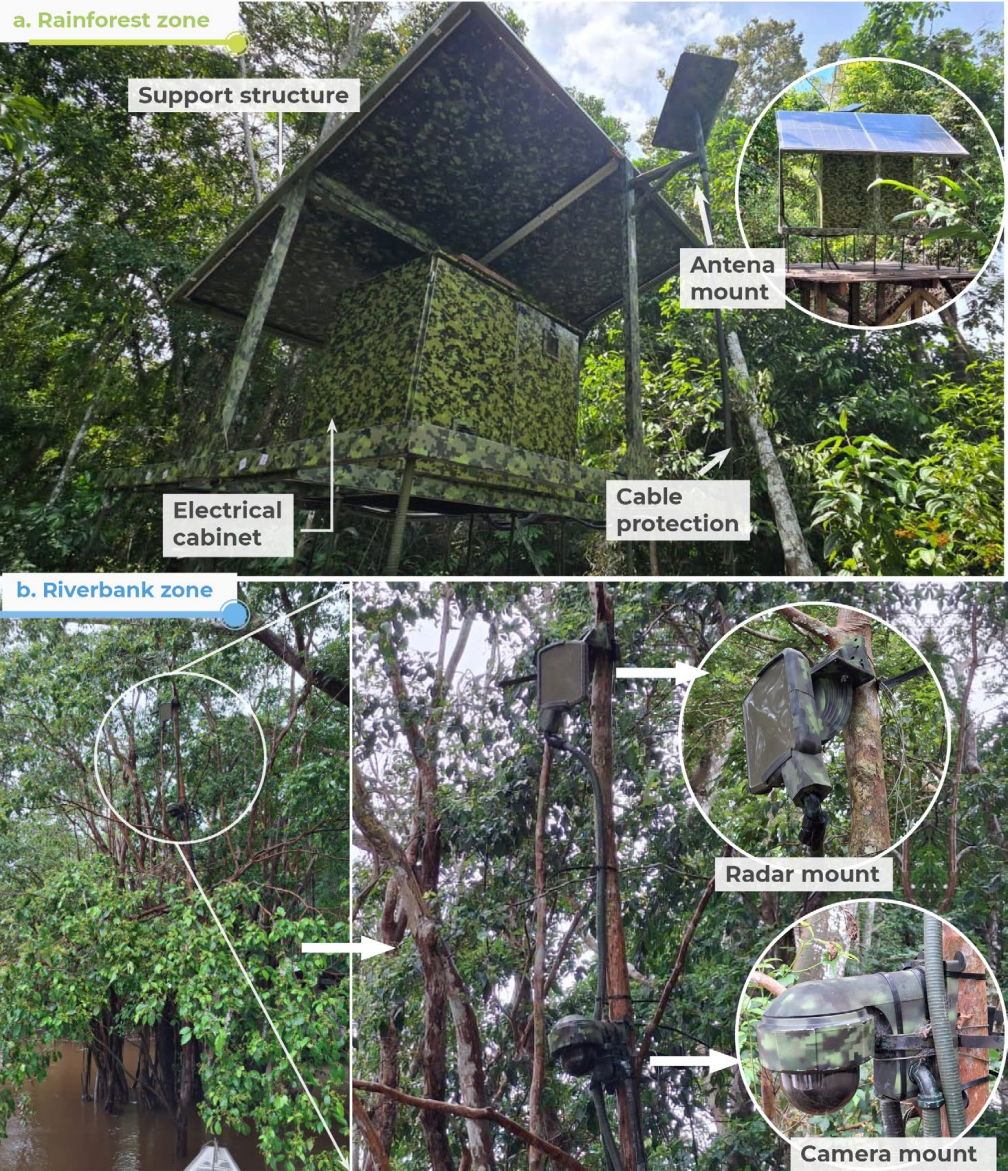
Installed Remote Monitoring System (RMS) prototype in Amacayacu National Natural Park (ANNP). Field photographs showing the final deployment configuration, including the rainforest zone (a) with the elevated support structure and photovoltaic modules, and the riverbank zone (b) with the tree‐mounted radar and camera system. The installation reflects the integration of enclosure design, corrosion protection, and camouflage strategies.

Once all the mounting and housing pieces were manufactured and coated, they were packed and transported from Bogotá D.C. to the ANNP. The RMS prototype was installed in December 2024 in ANNP, as shown in Figure 6.

For the rainforest zone (3°48′19.4″S 70°18′19.9″W), a site 70 m from the river was chosen, with foliage that provided good cover. Based on water marks, the maximum flood level was estimated, and a wooden platform was built to increase the height of the structure. A tall tree without thorns or insects was selected for the riverbank zone (3°48′20.0″S 70°18′21.3″W), with an optimal view of the river. Selective trimming was carried out in both zones to ensure the proper functioning of the radar, camera, antenna, and solar panels without affecting the ecosystem.

In the rainforest zone, the metal support structure with the solar panels was assembled and oriented according to the optimal sunlight direction. Then, the electrical cabinet and the antenna were installed. Simultaneously in the riverbank zone, the camera and radar were mounted and securely attached to the selected tree trunk. All cables were protected by inserting them in the conduits and connected. The cable protection was partially buried to minimize visual impact. Finally, the RMS was powered on and successfully tested on site. Waste materials were carefully removed to prevent any impact on the environment and transported for proper management.

## 4 Discussion

The prototype has been in operation at the ANNP since December 2024. During the first nine months, the system generated more than 10.000 alerts with target information and photos, allowing park rangers to visually confirm activity in the protected areas in real time. While the specific design solutions described in this study respond to the environmental and logistical conditions of the Colombian Amazon, several broader insights may be relevant to the deployment of technological monitoring systems in other conservation contexts where equipment must operate autonomously for extended periods. It is important to mention that at this stage, the present study focused on the technical performance, installation experience, and environmental robustness of the system. A quantitative evaluation of its effectiveness in supporting conservation monitoring or reducing illegal activities will require longer operational periods and dedicated ecological analyses.

This work proposes a comprehensive design methodology, addressing housing, mounting, and specialized coatings, for deploying a concrete technological solution in a complex environment. Further, this paper provides a documented validation experience that could serve as a practical roadmap for future implementations. Our approach is aligned with the priorities identified by Glover‐Kapfer et al. (2019) to develop conservation technology and with Berger‐Tal and Lahoz‐Monfort (2018), who emphasized the need for interdisciplinary collaboration with conservationists to tailor technological tools to the specific requirements of protected ecosystems. In the existing literature, we found works that were focused on particular technical aspects; for instance, (Miranda et al. 2000) provides insightful data on coatings and corrosion, and (Lascaro 1970) presents general advice concerning research and development for tropical environments. Regarding camouflage, various studies have suggested methodological approaches (Fiehler et al. 2007; Jacobs and Ausband 2018; Meek et al. 2022), but their strategies may harm the data quality (Meek et al. 2016). Concerning design, according to (Berger‐Tal and Lahoz‐Monfort 2018), many custom designs remain mere adaptations of military or healthcare equipment. These works frequently lack commercial recommendations or application to a specific case study. Considering the limitations identified in the mentioned previous studies, the following sections present the main lessons learnt of our work to help address and complement these gaps.

### 4.1 Installation and Design

Transporting and deploying the RMS required a coordinated team of 15 people, including RMS developers and park rangers. At ANNP, the local knowledge of park rangers was essential for selecting the installation zones. This was confirmed during the rainy season, when water levels rose by more than 7 m and the mounting and housing elements remained unaffected. However, the observed river level fluctuations suggest that positioning the camera and radar at a slightly greater height could potentially improve visibility under extreme conditions.

The three methods used to secure the mounts to the tree trunk at the riverbank zone performed effectively. However, small fastening components were found to be prone to loss during installation. This indicates that more stable, yet removable fastening alternatives could facilitate field deployment.

The interiors of the camera and radar mounts remained free of water and insects, and the rubber seals effectively prevented camera fogging. A slight tilt was observed in the camera mount, suggesting that reinforcement of the side piece may enhance structural stability. Although stainless‐steel screws were used, signs of oxidation were detected, indicating that alternative fastening materials, such as plastic‐based components, might be considered for future iterations.

Although the radar's original color blended well with the environment, its shiny surface reflects water and sunlight, increasing its visual detectability. This observation suggests that the use of anti‐reflective coatings could reduce visibility. However, such an approach would require careful evaluation, as it may influence the radar's sensitivity.

In the rainforest zone, the support structure, the antenna mount, and the electrical cabinet performed optimally. The modular design of the support structure facilitated transportation. However, the overall dimensions of the electrical cabinet (120 × 100 × 82 cm) made handling more difficult, indicating that dimensional optimization could improve future designs.

The protective conduits shielding the cables provided an effective barrier against environmental exposure, and the cables remained functional. However, the insertion of the cables into the conduits was time‐consuming, suggesting that alternative cable management solutions might enhance installation efficiency in future prototypes.

### 4.2 Performance Tests

The tests conducted to select the plastic materials and the coatings helped predict potential field failures and highlighted important insights concerning materials to withstand outdoors at tropical rainforest conditions. The ASA filament remained solid without signs of biodegradation or deterioration due to sunlight or water. The metallic pieces presented localized wear, particularly in the areas where coating was lost due to handling‐induced scratches during transportation. This reinforces the idea that a multilayer coating composed of anticorrosive paint, electrostatic paint, camouflage paint, and a varnish offers good protection against corrosion. As electrostatic paint offers a good barrier for protecting metal, it is unsuitable to create a camouflage appearance.

### 4.3 Camouflage

The selection of colors based on pixelated photographs and the print tests facilitated the final choice and highlighted the need for high contrast colors, to avoid a monochromatic appearance from a distance. The pixelated mottled pattern integrated effectively into the environment, although variations in the color of the riverbank logs suggest that alternative palettes should be explored in the future specifically for the elements attached to trunks.

The test prototype, including the designed mounting and housing elements and the achieved camouflage appearance, has enabled the continuous operation of the RMS since its installation. During this period, the system has not experienced any acts of vandalism, and all its components have remained operational.

## 5 Conclusion

The experience of designing and implementing the RMS in the Colombian Amazon highlights the potential of context‐driven design in engineering and demonstrates the technical feasibility of deploying autonomous monitoring infrastructure in remote tropical environments that may support future conservation monitoring initiatives. By addressing environmental conditions and infrastructure limitations, along with the appropriate selection of coatings, materials, and a camouflage strategy, this study demonstrates the feasibility of developing viable and replicable monitoring technologies. Beyond technical performance, the system has been positively received by park rangers, who describe it as “having eyes where it used to be impossible to have them.” Future work will assess long‐term ecological and operational impacts and explore its adaptation to other environmental contexts.

Beyond the specific deployment context, the design approach and lessons learned may contribute to the development of monitoring technologies for conservation programs operating in remote, humid, or infrastructure‐limited environments worldwide.

## Author Contributions

**María Barajas:** conceptualization (lead), investigation (lead), methodology (lead), validation (equal), visualization (equal), writing – original draft (lead). **Jorge Torres:** conceptualization (equal), funding acquisition (equal), investigation (equal), project administration (lead), validation (equal), writing – original draft (equal). **Paula Ortiz:** conceptualization (equal), investigation (equal), validation (equal), writing – original draft (equal). **Andrés Triana:** funding acquisition (equal), investigation (equal), project administration (equal), supervision (equal), writing – review and editing (equal). **Margarita Varon:** funding acquisition (lead), investigation (lead), project administration (lead), supervision (lead), writing – review and editing (lead).

## Funding

This study was partially supported by the “Sistema General de Regalías – Ministerio de Hacienda y Crédito Público–República de Colombia” through the project BPIN 2020000100431.

## Conflicts of Interest

The authors declare no conflicts of interest.

## Supporting information


**Data S1:** Supporting Information.

## Data Availability

The data for this study is in this manuscript and in the Supporting Information.
