# Supplementary material for: Enclosure and Camouflage Design of a Prototype Remote Monitoring System for the Protection and Conservation of Territories in the Colombian Amazon Rainforest
Source: Ecol Evol. 2026 Apr 20;16(4):e73491. doi: 10.1002/ece3.73491 (PMC13096577; doi:10.1002/ece3.73491)
Supplement: Supplementary file 1 — Data S1: Supporting Information. [file ECE3-16-e73491-s001.pdf]

**Empresa**	Alertas Radar Amazonas
**Nombre**	María Barajas
**Teléfono**	+57 310 3024467
**Correo electrónico**	alertasradar_nal@unal.edu.co

**Laboratorio**	Laboratorio de Electroquímica y Catálisis
**Ensayo**	Cámara Salina
**Orden de trabajo**	LQUI-035-2024

**Muestra**	
**Consecutivo**	LQUI-035-2024
**Cantidad**	15 muestras

**Ejecución del ensayo**	
**Fecha inicio**	13 de junio de 2024
**Fecha fin**	17 de junio de 2024

1. MÉTODO DE ENSAYO

**Preparación de las muestras**	N.A.
**Desviación adiciones o exclusiones de la norma o especificación**	N.A.

2. DESCRIPCIÓN DE LAS MUESTRAS

Muestra No.	Descripción	Observaciones iniciales
LQUI-035-2024- 1	Lámina cold-rolled	N.A.
LQUI-035-2024- 2	Lámina galvanizada	N.A.
LQUI-035-2024- 3	Lámina anticorrosivo	N.A.
LQUI-035-2024- 4	Lámina pintura electrostática	N.A.
LQUI-035-2024- 5	Lámina pintura grafiti	N.A.
LQUI-035-2024- 6	Lámina vinilo	N.A.
LQUI-035-2024- 7	Lámina hidro-impresión	N.A.
LQUI-035-2024- 8	Lámina epóxico	N.A.
LQUI-035-2024- 9	Lámina laca	N.A.
LQUI-035-2024- 10	Lámina poliéster	N.A.
LQUI-035-2024- 11	Lámina poliuretano	N.A.
LQUI-035-2024- 12	Lámina esmalte	N.A.

LQUI-035-2024- 13	Lámina grafiti en plástico	N.A.
LQUI-035-2024- 14	Lámina hidro-impresión en plástico	N.A.
LQUI-035-2024- 15	Hidro-impresión en tubo	N.A.

3. DATOS DEL ENSAYO

**Equipo**	Atlas SF corrosión exposure System
**Descripción del ensayo**	El ensayo de cámara salina determina la resistencia de un material a la corrosión sometiéndolo a un ambiente de extrema humedad, temperatura y químicamente activo. Las muestras son introducidas en una cámara donde una solución salina de cloruro de sodio al 5% en peso es atomizada como una niebla densa sobre las muestras continuamente a una temperatura constante de 35°C por min **100 horas**. El ensayo se realiza en el equipo Atlas SF corrosión exposure System y se basa en la norma ASTM B-117. El ensayo es aplicado para determinar la vida útil de los revestimientos en los metales
**Reactivos**	Cloruro de sodio y agua
**Normas o especificación aplicable**	ASTM B-117
**Contramuestra**	N.A.

4. RESULTADOS

Registro fotográfico: Se anexan los registros fotográficos de las pruebas realizadas.

Muestra	0 hr.	100 hr.
LQUI-035-2024- 1	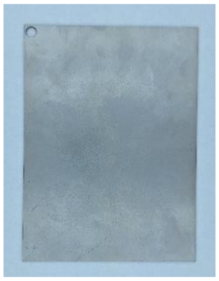	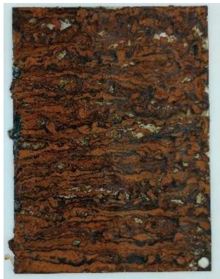
LQUI-035-2024- 2		

	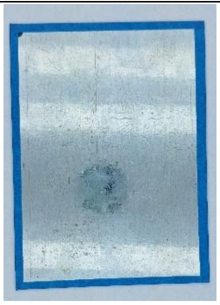	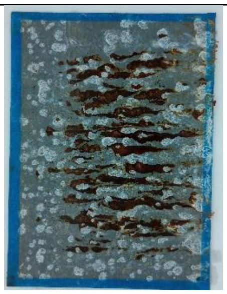
LQUI-035-2024- 3	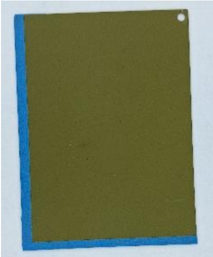	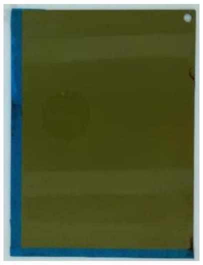
LQUI-035-2024- 4	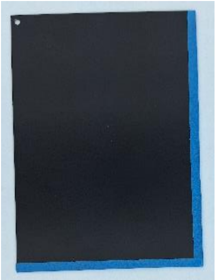	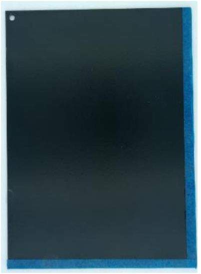
LQUI-035-2024- 5	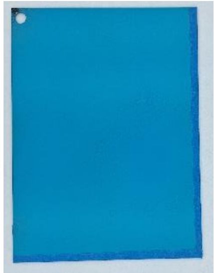	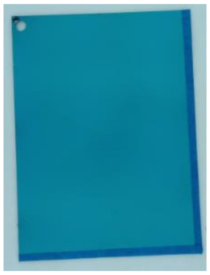
LQUI-035-2024- 6		

	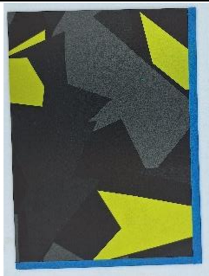	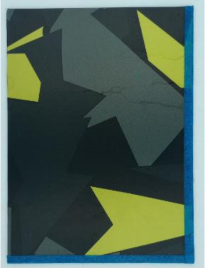
LQUI-035-2024- 7	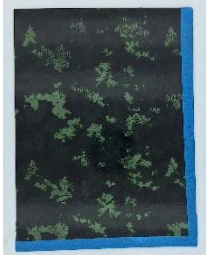	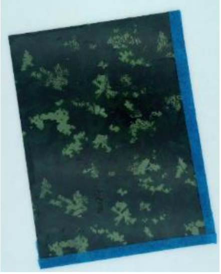
LQUI-035-2024- 8	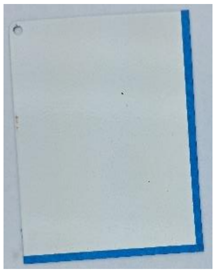	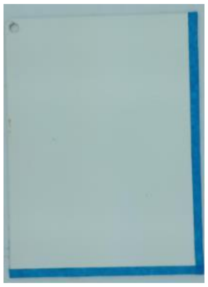
LQUI-035-2024- 9	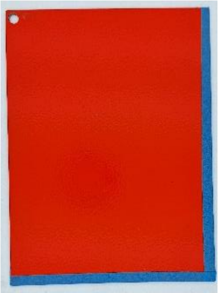	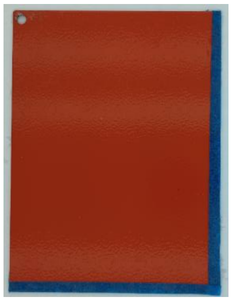
LQUI-035-2024- 10	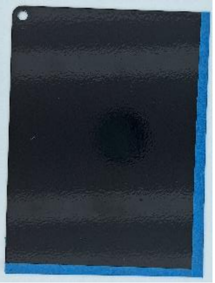	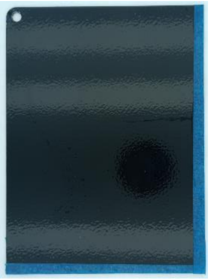

LQUI-035-2024- 11	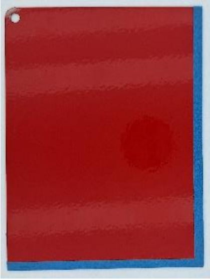	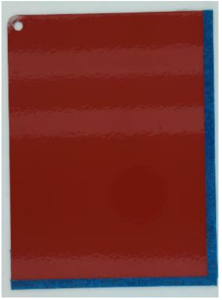
LQUI-035-2024- 12	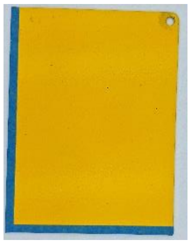	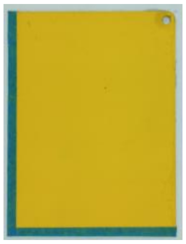
LQUI-035-2024- 13	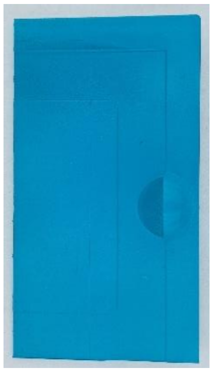	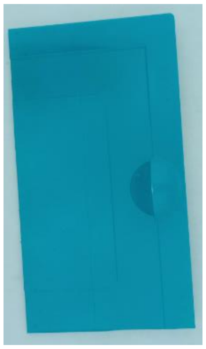
LQUI-035-2024- 14		

	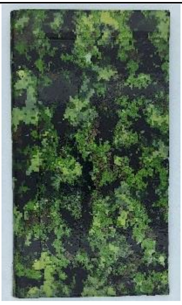	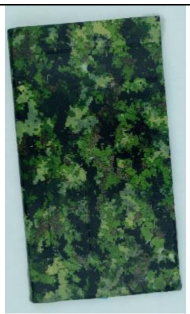
LQUI-035-2024- 15	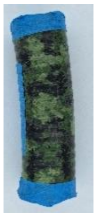	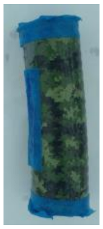

Mario Noriega

MARIO ANDRÉS NORIEGA VALENCIA
Coordinador LIQ
